# Modulators of protein–protein interactions as antimicrobial agents

**DOI:** 10.1039/d0cb00205d

**Published:** 2021-02-03

**Authors:** Rashi Kahan, Dennis J. Worm, Guilherme V. de Castro, Simon Ng, Anna Barnard

**Affiliations:** Department of Chemistry, Molecular Sciences Research Hub, Imperial College London 82 Wood Lane London W12 0BZ UK a.barnard@imperial.ac.uk

## Abstract

Protein–Protein interactions (PPIs) are involved in a myriad of cellular processes in all living organisms and the modulation of PPIs is already under investigation for the development of new drugs targeting cancers, autoimmune diseases and viruses. PPIs are also involved in the regulation of vital functions in bacteria and, therefore, targeting bacterial PPIs offers an attractive strategy for the development of antibiotics with novel modes of action. The latter are urgently needed to tackle multidrug-resistant and multidrug-tolerant bacteria. In this review, we describe recent developments in the modulation of PPIs in pathogenic bacteria for antibiotic development, including advanced small molecule and peptide inhibitors acting on bacterial PPIs involved in division, replication and transcription, outer membrane protein biogenesis, with an additional focus on toxin–antitoxin systems as upcoming drug targets.

## Abstract 2

This review describes recent efforts towards the modulation of protein–protein interactions in infectious bacteria.

## Graphical abstract

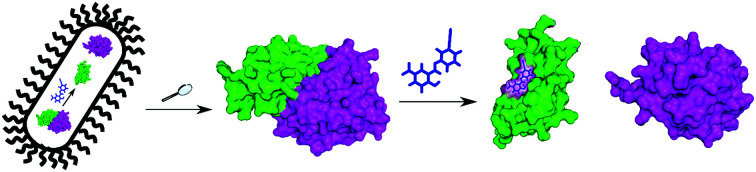

## 1 Introduction

Antibiotic resistance and the recurrence of bacterial infections are two of the most serious threats to future global public health, with the potential to affect anyone in any country in the world. The Centre of Disease Control (CDC) estimates that 2.8 million people acquire an antibiotic-resistant infection in the US every year, already resulting in more than 35 000 fatalities.^1^ Without effective antibacterial compounds, infections by multi-drug resistant or tolerant bacteria could result in life-threatening conditions in people with minor injuries. In hospital settings, recalcitrant infections compromise the success of surgery and the therapy of immunosuppressed patients. In addition, the health care costs for patients with resistant infections are significantly higher than for other patients, due to obligatory isolation, longer hospital stays and the use of more expensive drugs.^2^

Although antibiotic treatment is naturally linked to the occurrence of resistance in bacteria, misuse in humans and animals has accelerated the generation of antibiotic-resistant bacterial strains around the globe. Furthermore, the discovery of innovative anti-infective agents has been sparse, also attributed to the lack of investment in antimicrobial research by major pharmaceutical companies.^3^ Most of the antibiotics marketed in the last decades are based on existing drugs, which aim to overcome the resistance acquired by bacteria against their parent compounds.^4^ However, derivatisation of existing drug scaffolds that are acting on already targeted bacterial cell machineries risks rapid emergence of novel resistance mechanisms, requiring a constant compound optimization.

The major challenge associated with recalcitrant infection is the ability of bacteria to evade antibiotic treatment by three different mechanisms: resistance, tolerance and persistence. While resistance is acquired on the genetic level, allowing the bacterial population to grow in the presence of the antibiotic, tolerance and persistence enable bacteria to transiently survive antibiotic exposure.^5^ The distinction between the latter two being that tolerance is homogenously displayed by the whole bacterial population, while persistence describes the formation of a subpopulation of bacteria able to survive antibiotic treatment.^5^

Current antibiotics mostly act on classical targets such as bacterial enzymes, ribosomal RNA, cell wall construction and cell membrane function. It is clear, however, that antibacterial agents with innovative modes of action are urgently needed to tackle mechanisms leading to treatment failure, and to explore unconventional paths in the fight against resistant and recurrent infections. Bacterial protein–protein interactions (PPIs) are highly promising and, thus far, underexplored targets for antibiotic drug discovery.

### 1.1 Targeting PPIs

PPIs have, until recently, been considered unsuitable drug targets, due to their large, flat interaction surfaces (∼1000–2000 Å^2^).^6^ There is however growing research that refutes this notion, and PPIs are now being investigated as targets for novel therapeutics.^7^ A subset of the amino acid residues involved in the formation of a PPI, known as the hotspots, primarily drive their formation and confer the majority of their binding energy,^8^ making the challenge of developing targeting molecules less daunting.

Various techniques have been utilised to identify lead compounds that can target PPIs. Structurally diverse small molecule compounds have been identified by traditional high throughput screening (HTS), fragment-based lead discovery and also structure-based rational design, as some PPIs have small-molecule sized patches of around 250–900 Å^2^ formed by clusters of hotspots.^9,10^ Computational methods such as virtual screening, *in silico* experiments and homology modelling are frequently used to aid the design of PPI-targeting small molecules.^11^ Peptides offer the possibility to cover larger surface areas and are often developed into cyclic variants or peptidomimetic compounds to achieve more drug-like properties.^12–14^ Furthermore, HTS methods using linear and macrocyclic peptide libraries are increasingly used to identify novel PPI inhibitors.^15^

Targeting PPIs has already yielded new treatments for cancer. Trials for idasanutlin, which targets the MDM2–p53 interaction, and apabetalone, which targets bromodomains, are underway for cancer treatments.^16^ Venetoclax, which is prescribed to treat chronic lymphocytic leukaemia (CLL), is a BH3-mimetic, resulting in apoptosis of CLL cells due to inhibition of BCL-2 activity.^17^ Furthermore, stapled peptides targeting MCL-1 protein, a member of the BCL-2 family, are being developed as apoptosis inducers in cancer cells^18^ and ALRN-6924, a stapled peptide which targets both MDM2 and MDMX, entered clinical trials in 2017 with promising initial results.^19^

### 1.2 PPIs in infection

Many crucial cellular functions in viruses, bacteria and other pathogens rely on PPIs. The interactions can be pathogen–pathogen, host–pathogen or host–host, all of which are interesting targets for novel anti-infective agents. Targeting host–pathogen interactions has been successful, with drugs such as antiretroviral drugs enfuvirtide and maraviroc for the treatment of HIV/AIDS.^20^

Many PPIs are linked to bacterial processes absent in eukaryotic cells, making them attractive targets for the discovery of new pathogen-selective leads. The bacterial interactome has the potential to enable the identification of numerous targets for novel therapies.^21^ As PPIs are linked to crucial processes within bacteria, disrupting these interactions can lead to inhibition of bacterial cell growth and/or result in cell death. Many PPIs have already been identified and targeted in pathogenic bacteria. These are involved in a wide range of vital functions, such as bacterial division (FtsZ and SSBs), transcription (RNA polymerases) and toxin–antitoxin pairs whereby toxins have been implicated in programmed cell death as well as persistence. Both small molecule and peptide inhibitors have been developed and multiple screening techniques have been applied for their identification.

This review focuses on PPIs relating directly to functions in bacteria, *i.e.* pathogen–pathogen interactions. We present an overview of bacterial PPIs that have been identified as anti-infective targets and the approaches used to identify and progress small molecules and peptides targeting different classes of interaction, highlighting the most promising compounds currently in development.

## 2 PPIs as antibacterial targets

### 2.1 Targeting division and replication

Because of their essential role in sustaining cell viability, multiple members of the DNA replication and cell division machinery have been targeted for antimicrobial therapies.^6,16,22^ Despite efforts described in the literature, inhibitors of components of topoisomerase II represent the only marketed drugs targeting this class of proteins.^22^ These antibiotics can be divided in two groups: inhibitors of the ATPase site of DNA gyrase, that can effectively block the relaxation of supercoiled DNA (*e.g.* novobiocin);^23,24^ and stabilisers of the complex between cleaved DNA strands and topoisomerase II, which are capable of triggering cell death via the accumulation of DNA fragments (*e.g.* ciprofloxacin).^25^ Although these compounds are amongst the most widely used antibiotics in the clinic, many limitations of their administration have been identified in recent years. The development of novel aminocoumarins (typical DNA gyrase inhibitors) is often restricted by their poor drug-like physicochemical properties.^24^ On the other hand, the widespread use fluoroquinolones (well-characterised inducers of fragmented DNA build-up) to treat multiple infections has caused genetically resistant strains to arise. Fluoroquinolone-resistant strains of *Campylobacter* and *Salmonella* spp. were highlighted by the WHO as high-priority targets for antimicrobial research.^26^

Due to the multi-protein complexity of the replisome and the divisome, and the poor conservation with eukaryotic homologs, PPIs have been explored as attractive routes for the development of inhibitors of cell division and DNA replication. Notably, three approaches have been investigated in recent years: (i) disruption of the PPI between FtsZ (filamenting temperature-sensitive protein Z) and ZipA (FtsZ interacting protein A), (ii) interference with the PPI network of SSB (single-stranded DNA-binding protein) and (iii) prevention of the recruitment of essential enzymes by the β-subunit of DNA polymerase III (β-sliding clamp) during replication. Reviews describing small-molecule development for each of these approaches can be found elsewhere,^6,16,22,27^ therefore focus will be given to the key findings for these strategies.

### 2.1.i FtsZ–ZipA inhibition

**Fig. 1:**
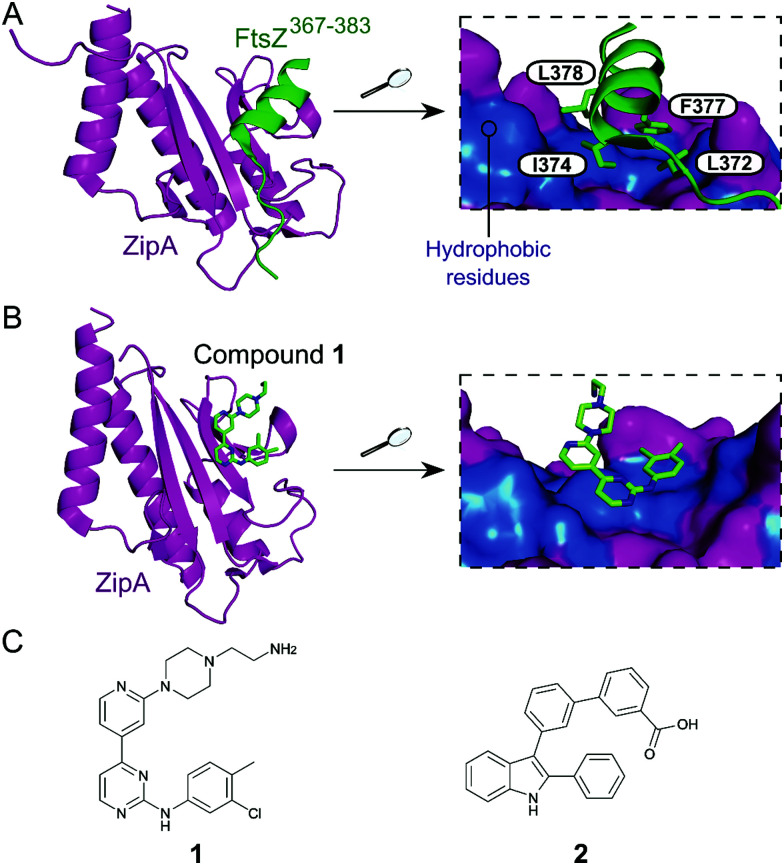
(A) Crystal structure (PDB: 1F47)^29^ of the C-terminus of *E. coli* FtsZ (green) bound to *E. coli* ZipA (magenta). The interaction is driven by a solvent exposed hydrophobic patch (blue surface, right chart) present in ZipA. (B) Crystal structure (PDB: 1Y2F)^31^ of inhibitor **1** bound to *E. coli* ZipA. The compound interacts at the same site as FtsZ (right chart). (C) Chemical structures of FtsZ–ZipA PPI inhibitors.

Inhibition of the FtsZ–ZipA contact is so far the most advanced strategy targeting cell division PPIs. FtsZ is a tubulin homolog responsible for the formation of a ring-like structure (Z-ring) that acts as a scaffold for the assembly of a number of proteins. ZipA bridges FtsZ and the bacterial membrane, allowing the Z-ring to guide the synthesis and reshaping of the peptidoglycan wall during division. Therefore, inhibition of this PPI would lead to incomplete division, subsequently triggering bacterial cell death.^28^ The C-terminal α-helix of FtsZ was identified as the key region of interaction with a large hydrophobic surface on ZipA (Fig. 1A).^29^

Kenny *et al.* employed a high-throughput fluorescence polarisation (FP) assay to identify competitors of the interaction between ZipA and a 17-mer C-terminal FtsZ peptide (*K*^ZipA^_d_ = 7 μM). Compound **1** (Fig. 1B and C) was selected as the most active hit (*K*_i_ = 12 μM) and confirmed to bind to ZipA on the expected PPI interface.^30^ Despite the promising start, high cytotoxicity in *Candida albicans* indicated that **1** could also harm eukaryotic cells.^31^ In order to overcome this issue, multiple follow-up studies introducing novel scaffolds via fragment screening and computational methods were performed, establishing structure–activity relationships (SAR).^30–34^ A fragment-merging approach led to compound **2** (Fig. 1C), which possessed a moderate MIC (10–100 μM) against many bacterial pathogens (*e.g.* MIC *Escherichia coli* imp = 20 μM) and promisingly no toxicity to *C. albicans* (MIC > 300 μM). Two-dimensional NMR experiments confirmed binding to the expected site and an IC_50_ of 192 μM for inhibition of the PPI was determined by FP.^32^ The discrepancy between PPI inhibition and activity indicates that further mode-of-action studies are required before progressing the series forward. As MIC values were considerably lower than the IC_50_ (determined at non-limiting ZipA concentration of 500 nM), **2** and related compounds might act by additional unknown mechanisms beyond ZipA-FtsZ inhibition.

#### 2.1.ii Single-stranded DNA-binding protein (SSB)

Multiple factors support the development of SSB mimetics as antimicrobials targeting DNA replication. SSB is essential for replication to progress without stalling by specifically binding single-stranded DNA.^35^ This interaction protects the genome from nuclease damage, helps to keep the strands separated while the daughter DNA is synthesised and allows the recruitment of essential enzymes. The latter is mediated by a C-terminal (Ct) linear sequence of six to nine highly conserved residues.^36^ Mimicking this motif with small molecule inhibitors has great therapeutic potential, as resistance mutations on SSB would require complementary mutations amongst all of its binding partners.

**Fig. 2:**
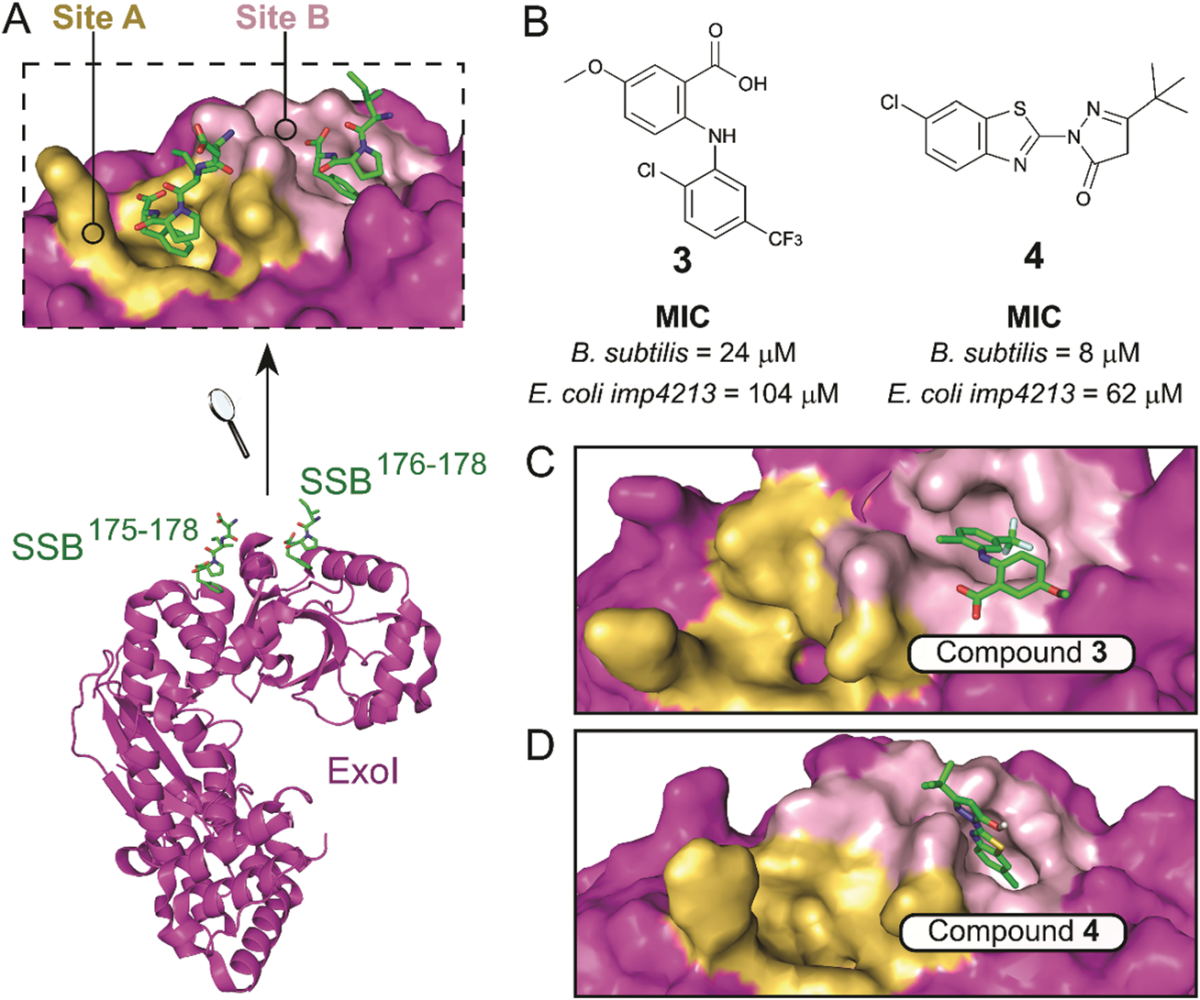
(A) Crystal structure (PDB: 3C94)^37^ of *E. coli* ExoI (magenta) bound to a *E. coli* SSB Ct peptide (green). ExoI can interact simultaneously with two peptide molecules via the binding sites A (yellow surface) and B (pink surface), as shown in the upper chart. (B) Chemical structures and activity of inhibitors **3** and **4**. The *E. coli* ExoI-bound crystal structures of inhibitors **3** (PDB: 3HP9)^38^ and **4** (PDB: 3HL8)^38^ are shown in (C) and (D), respectively.

The exonuclease I (ExoI) was identified early on as a promising SSB-interacting protein to target due to its well-characterised role in DNA repair during replication.^37^ Disruptive mutations of the ExoI–SSB contact were shown to be lethal to *E. coli*, validating the relevance of targeting this PPI. The co-crystal structure of ExoI in complex with a SSB Ct peptide (Fig. 2A) revealed two independent binding sites (commonly referred as sites A and B). Site B was identified as essential for SSB-mediated ExoI activity, therefore formed the main focus for the development of PPI inhibitors.^37^ Keck *et al.* performed an HTS by FP and identified four promising hits able to displace an *E*. *coli* SSB mimetic peptide (Fluorescein-WMDFDDDIPF-COOH, *K*^ExoI^_d_ = 136 nM) with an IC_50_ below 100 μM.^37–39^ Co-crystal structures of ExoI in complex with two of these compounds (**3** and **4** shown in Fig. 2B and C, respectively) confirmed they were binding to the SSB site B. Additionally, these compounds possessed a growth inhibitory effect on a large panel of Gram-positive and Gram-negative bacteria, without demonstrating any toxicity to the eukaryotic *Saccharomyces cerevisiae*.

Beyond ExoI, the inhibition of other SSB binding partners has also been investigated: (i) a fragment screening on the DNA primase DnaG with a combination of protein- and ligand-observed NMR experiments;^40^ (ii) HTS of the helicase PriA via AlphaScreen.^41^ Interestingly, due to the promiscuous nature of the SSB C-terminal tail, selected hits from these independent studies were found to bind to multiple SSB binding partners, indicating the potential for pan-selective inhibition of the SSB interactome.^40,41^

#### 2.1.iii β-Sliding clamp

The β-sliding clamp, like the SSBs, is an essential replication protein capable of interacting with multiple enzymes. In this case, the β-sliding clamp can recognise a small epitope of up to nine residues present in its binding partners. It can establish a PPI with important enzymes, such as DNA polymerases (I, II, IIIα, IIIδ, IV and V) and DNA ligase, bringing them to the DNA strand while the protein clamp complex moves along the genetic sequence.^42^ The lack of conservation with the eukaryotic homolog PCNA (proliferating cell nuclear antigen) makes this interaction an interesting region for inhibitor development.^43^

**Fig. 3:**
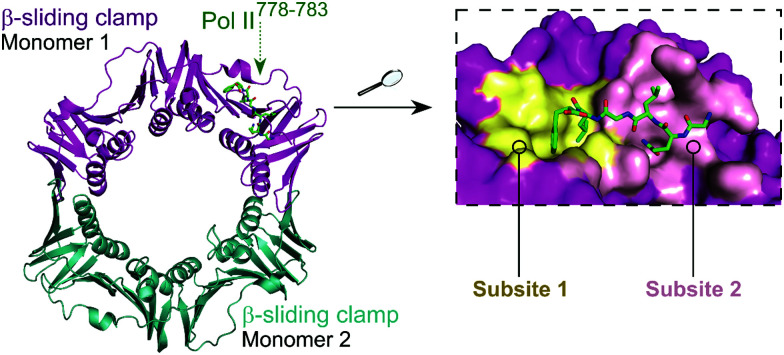
Crystal structure (PDB: 3D1E)^46^ of the *E. coli* β-sliding clamp dimer (one monomer is shown in magenta and the second monomer is shown in cyan) bound to the *E. coli* Pol II C-terminus peptide (green). The binding site is divided into two subsites (1 and 2, shown in yellow and pink, respectively) with the Pol II peptide extending over both regions (right chart).

**Fig. 4:**
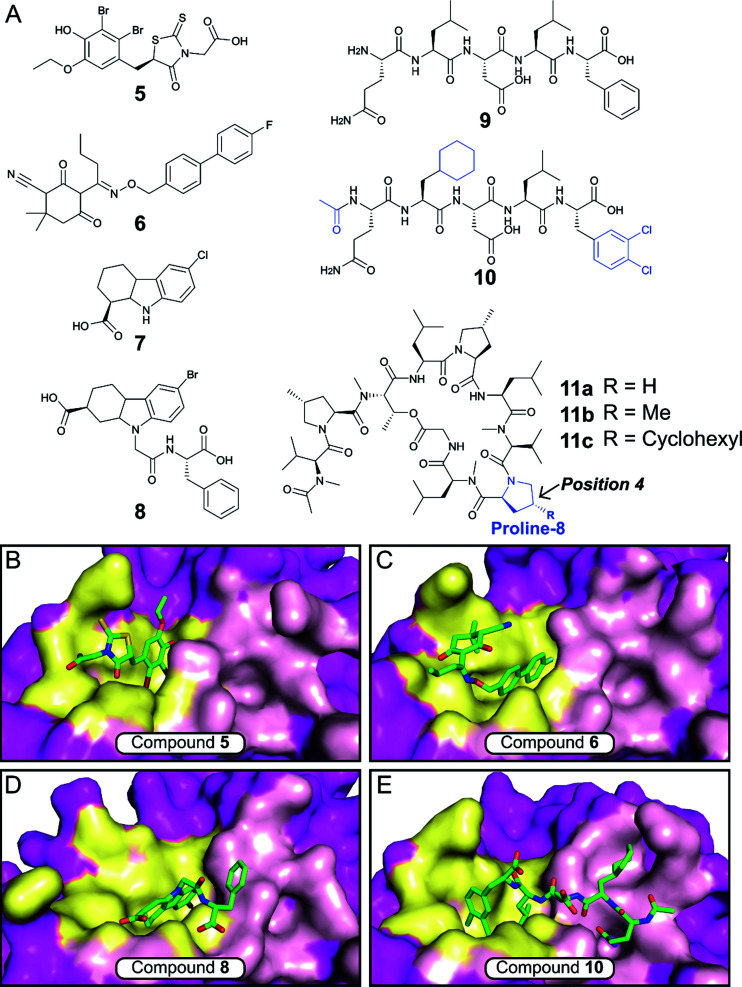
(A) Chemical structures of small molecule and peptidic inhibitors of the β-sliding clamp PPI site. The crystal structures of **5**, **6**, **8** and **10** bound to the *E. coli* β-sliding clamp (PDB accession codes 3D1G,^46^3QSB,^47^4PNU^48^ and 3Q4L,^50^ respectively) are shown in (B), (C), (D) and (E), respectively. Subsites 1 and 2 are shown as yellow and pink surface, respectively, and the remaining regions are shown as magenta surface.

The *E. coli* β-sliding clamp specifically recognises proteins carrying a linear QL[S/D]LF consensus sequence. Crystal structures with peptides mimicking the DNA polymerase II, III and IV (Pol II, Pol III and Pol IV, respectively) revealed the interactions to be highly hydrophobic and divided into two subsites (Fig. 3).^44–46^ In the search for inhibitors of specific DNA polymerase interactions with the clamp complex, Georgescu *et al.* performed a HTS using FP,^46^ based on the displacement of a fluorescent Pol III 20-mer peptide (*K*_d_ = 2.7 μM). Compound **5** (Fig. 4A) was selected as a promising hit (*K*_i_ = 10 μM) with good activity in replication assays, and a co-crystal structure (Fig. 4B) revealed interactions with subsite 1 of the pocket. However, the presence of the promiscuous rhodamine core in the structure and poor antibacterial activity held back further development of this series.

Nevertheless, this work motivated multiple follow-up studies that were able to identify novel scaffolds for inhibition at the same site. Compound library filters for structures with similar properties to the subsite 1 binding motif (consensus sequence residues DLF) identified five novel inhibitors (IC_50_ ≈ 40–400 μM) that did not possess the rhodamine ring (*e.g.* biaryl oxime **6**, Fig. 4A).^47^ Fragment-based screening via computational docking and FP also introduced a 1,2,3,4-tetrahydrocarbazole core (**7**, Fig. 4A) with good *in vitro* activity (IC_50_ = 115 μM) and confirmed binding to subsite 1.^48^ Additionally, fragment **7** demonstrated reasonable MIC (40–80 μM) against a range of Gram-negative and Gram-positive bacteria.^48,49^ SAR studies ultimately led to compound **8** (Fig. 4A), where the fragment core was extended by a phenylalanine motif to make further interactions in the pocket (Fig. 4D).^49^ Compound **8** possessed both improved affinity to the β-sliding clamp (*K*_i_ = 15 μM) and inhibition of DNA replication (IC_50_ = 31 μM), although no antimicrobial data was reported.

Besides small-molecule inhibition, potent peptide inhibitors of the β-sliding clamp PPI hub have also been identified (Fig. 4A).^47,50,51^ Structure-guided optimisation of the pentapeptide consensus sequence **9** using surface plasmon resonance (SPR) (IC^SPR^_50_ = 12.4 μM) led to compound **10** (IC^SPR^_50_ = 77 nM), a potent binder that covers both subsites of the pocket (Fig. 4B). The acetylation of the terminal glutamine residue establishes a hydrogen bond with the backbone of arginine-365 of the protein. The introduction of the hydrophobic sidechains of cyclohexyl-l-alanyl and 3,4-dichlorophenylalanine provided a further increase in affinity, with the latter presenting the highest contribution in the stability of the peptide–protein complex.

Interestingly, studies on the bactericidal cyclic peptide griselimycin (**11a**, Fig. 4A) revealed this class of natural products to act by inhibiting the PPI site of the β-sliding clamp.^52^ Despite its antibacterial activity being known for more than 60 years,^53^ the poor metabolic stability of griselimycin hindered its development. However, its potent activity against drug-resistant *Mycobacterium tuberculosis* (Table 1) and disclosed mode of action has revived the interest of many researchers. Proline-8 was identified as the main labile site responsible for the degradation of **11a***in vivo*. Insertion of a single methyl group at position 4 of the pyrrole ring (**11b**) significantly increased microsomal stability (Table 1). The addition of a cyclohexyl group at the same position (**11c**) provided stability and higher lipophilicity, boosting the antibacterial activity.^52^

**Table 1:** Activity and stability data of griselimycin-based β-sliding clamp PPI inhibitors

Compound	*K* ^SPR^_d_ (*M. tuberculosis* clamp) [pM]	Metabolic stability^a^ [%]	Oral bioavailability [%]	MIC (*M. tuberculosis*) [μM]
**11a**	100 ± 81	62	48	0.90
**11b**	110 ± 11	100	47	0.53
**11c**	200 ± 79	86	89	0.05

a Percentage remaining in liver microsomes after 20 min.

### 2.2 Targeting bacterial transcription

Transcription is an essential cellular process involving the synthesis of RNA from a genomic DNA template, either as mRNA that is translated into proteins or as rRNA that is assembled into the 70S ribosome. Rifamycin and fidaxomicin are the only marketed antibiotics that act on bacterial transcription. The former targets the active site in bacterial RNA polymerase (RNAP) while the latter targets the RNAP clamp and both are highly susceptible to resistance.^54,55^ Since a number of transcription factors form PPIs that regulate all aspects of transcription in bacteria, these interactions are promising targets for the development of novel antibiotics.

### 2.2.i RNA polymerase (RNAP)

**Fig. 5:**
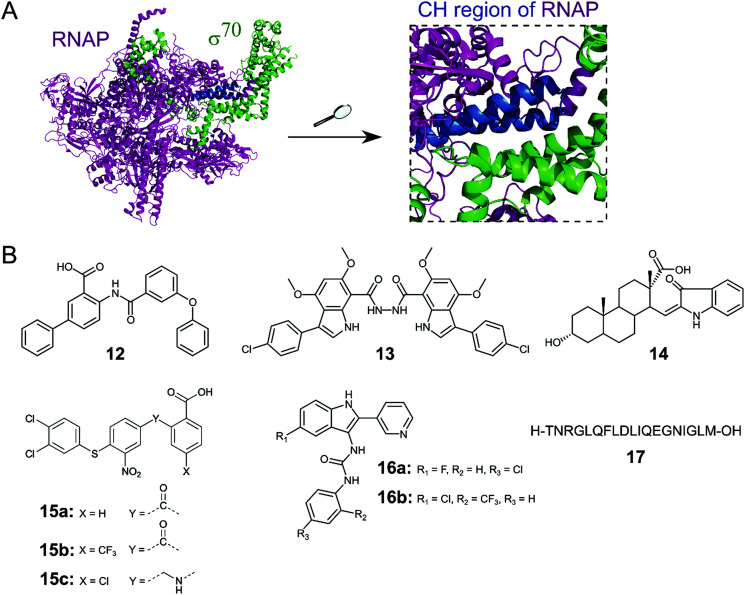
(A) Crystal structure of *E. coli* RNAP holoenzyme (PDB: 4LJZ)^62^ with RNAP core enzyme coloured in magenta and *σ*^70^ coloured in green. Enlarged picture (right chart) shows the critical interaction of the β′ clamp-helix (CH) region (blue) of RNAP with the N-terminal domain of *σ*^70^. (B) Small molecule and peptide inhibitors of RNAP–*σ* PPI.

Initiation of transcription of RNA in bacteria requires the association of the RNAP core enzyme (2α, β, β′ and ω subunits) with the transcription factor Sigma (*σ*) to form the RNAP holoenzyme. The upstream interaction with *σ* increases the specificity of RNAP for promotor regions, enabling transcription to start at the correct sites.^56^ Both the RNAP core and *σ* transcription factors are conserved across Gram-negative and Gram-positive bacterial strains and the main *σ* factors (termed *σ*^70^ in Gram-negative and *σ*^A^ in Gram-positive bacteria) do not have direct eukaryotic homologs,^57^ making potential inhibitors of this PPI promising broad spectrum antibacterial agents. Although RNAP and *σ* interact via an extensive network of contacts between regions 1–4 in *σ* and RNAP subunits β and β′, mutagenesis studies have identified hot-spot residues in the β′ clamp-helix (CH) region of RNAP and the small *σ*_2.2_ region that contribute most of the binding enthalpy.^58,59^ The CH region of RNAP consists of two antiparallel α-helices connected by a loop (Fig. 5A) and forms multiple electrostatic and hydrophobic contacts with *σ*_2.2_, therefore, disruption of this binding interface enables PPI inhibition.^60^

Several small molecule inhibitors of the RNAP–*σ* interaction have been identified. Hinsberger *et al.* used a pharmacophore model based on the alignment of known RNAP inhibitors for the screening of a 2000-compound in-house library.^61^ From 64 hits, 11 compounds were confirmed to be transcription inhibitors and three compounds were chosen for derivatisation to improve inhibitory activity. The best compound **12** (Fig. 5B) exhibited an IC_50_ of 13 μM in an RNAP transcription assay and disruption of the PPI between the RNAP β′ subunit and *σ*^70^ as mode of action for **12** was confirmed in an ELISA-based RNAP assembly assay (IC_50_: 47 μM). Furthermore, **12** displayed good antibacterial activity against Gram-positive *Bacillus subtilis* and *Staphylococcus aureus* with MICs of approximately 7 and 15 μM, respectively, but activity against Gram-negative *E. coli* K12 and *Pseudomonas aeruginosa* was low.^61^

The Lewis group has published several studies on novel RNAP–*σ* inhibitors. Key residues from *σ*^A^_2.2_ that are required for interaction with the β′-CH region were used for the generation of a pharmacophore model.^60^ With this model, a novel in-house library of peptidomimetic compounds was screened and bis-indole **13** (Fig. 5B) was further characterized. Compound **13** was found to bind to the β′-CH region and inhibit RNAP–*σ*^A^-mediated transcription *in vitro* with a *K*_i_ of 6 nM. Growth inhibition of cultured Gram-negative *E. coli* and Gram-positive *S. aureus* USA300 could be observed, but only at very high concentrations of **13** (>1 mM), suggesting that cellular penetration of **13** is likely to be poor.^60^ In order to obtain smaller β′–CH–*σ* inhibitors with potentially enhanced cellular permeability, 39 new small molecules based on mono-indole and mono-benzofuran scaffolds were synthesized.^63^ The new molecules displayed lower potencies in an ELISA-based β′–*σ*^A^ inhibition assay compared to the previously described bis-indoles, but higher antibacterial activities against *B. subtilis* and/or *E. coli* in a growth inhibition assay were observed for several compounds.^63^

In a study by Ma *et al.*, a more comprehensive pharmacophore model for the β′–CH–*σ*_2.2_ interaction was established and the model was used to screen the mini-Maybridge library (53 000 compounds).^64^ Out of 27 hits, **14** (Fig. 5B) was found to be the most active compound. In a RNAP transcription assay, **14** exhibited an IC_50_ of around 50 nM. Inhibition of the RNAP β′–*σ*^A^ PPI by **14** was confirmed by a competitive ELISA and **14** demonstrated specificity for bacterial RNAP. Compound **14** also showed promising antibacterial activity against Gram-positive *B. subtilis* with a MIC of <50 μM, however, activity against Gram-negative *S. aureus* strain USA300 was much lower with a MIC of around 94 μM.^64^ A second hit compound from the described screen was used in a recent study to generate a small series of derivatives.^65^ Out of the new series, **15a** (Fig. 5B) was found to be the most interesting, with a higher β′–CH–*σ*^A^ inhibitory activity than the parent compound and promising antibacterial activity against different Gram-positive cocci (*Streptococcus pyogenes*, *Streptococcus agalactiae* and *Staphylococcus epidermidis*) with MICs of 36 μM.^65^ Two structural optimization studies of triaryl compound **15a** were reported very recently. *para*-Substitution of the benzoic acid ring of **15a** with a trifluoromethyl group (**15b**) in the first study led to a compound with micromolar β′–CH–*σ*_2.2_ inhibitory activity and strong antibacterial activity against *S. epidermidis* with a MIC of 1 μM.^66^ In the second study, substitution of the carbonyl linker of **15a** with a methylamine linker in combination with *para*-substitution of the benzoic acid with a chlorine moiety led to **15c** with high activity against *Streptococcus pneumoniae* and *S. pyogenes* (MICs of 2 μM), comparable to vancomycin.^67^

Sartini *et al.* developed and optimized a bioluminescence resonance energy transfer (BRET) assay in yeast as a novel screening platform for the discovery of RNAP β′–*σ*^70^ inhibitors.^68^ Using their assay, 5000 compounds, *in silico* selected from a larger 34 000 compound library, were screened and 7 hits were obtained. Two indol-3-yl-urea derivatives (**16a**/**b**) of one hit compound were further investigated and found to preferentially bind to the RNAP β′ subunit and inhibit RNAP transcription *in vitro* with IC_50_ values of around 30 μM. The two derivatives **16a**/**b** also exhibited antibacterial activity against Gram-positive *B. subtilis*, *S. aureus* and *Listeria monocytogenes* with MIC values in the range of around 20–100 μM.^68^

Two studies have described peptides targeting the RNAP–*σ* interaction. Hüsecken *et al.* rationally designed 16 peptides covering different regions of the *E. coli σ*^70^:core interface and examined them as potential PPI inhibitors.^69^ Peptide **17** (Fig. 5B), derived from the *σ*^70^_2.2_ region, was found to be the most active compound with an IC_50_ of 5 μM in a RNAP transcription inhibition assay. Structural investigation by molecular dynamics simulation combined with mutagenesis studies revealed that **17** binds to the β′–CH region and also interacts with the β′ lid–rudder-system, with both sites being important for potent PPI inhibition. A recent study attempted to further optimize peptide **17**.^70^ An alanine scan of **17** was performed to identify the core sequence and stapled variants of **17** using copper-catalysed azide–alkyne cycloaddition were synthesized with the aim of improving inhibitory potency. However, despite an increase in α-helicity, stapling of **17** almost completely abolished its inhibitory activity.^70^

### 2.2.ii N-utilization substances (Nus) B and E

**Fig. 6:**
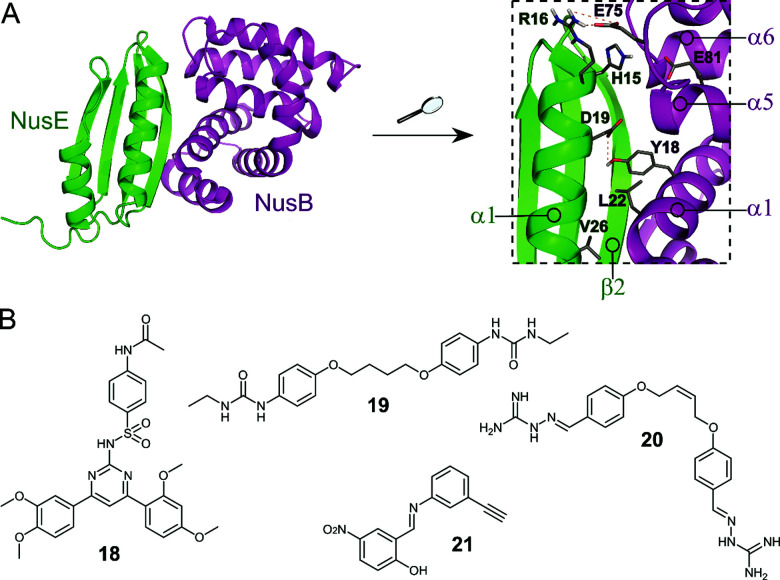
(A) Crystal structure of the *E. coli* NusB–NusE complex (PDB: 3D3B)^77^ with NusE coloured in green and NusB in magenta. Enlarged picture (right chart) shows the NusB–NusE PPI interface with residues (shown as sticks) H15, R16, D19 and V26 of the α1-helix of NusE interacting with Y18, L22, E75 and E81 of NusB; key hydrogen bonds are indicated by dashed orange lines. (B) Small molecule inhibitors of the NusB–NusE PPI.

The Nus transcription factors are conserved across bacterial species and are essential for viability. The proteins NusA, NusB, NusE, NusG and SuhB form the Nus factor complex, which interacts with rRNA and RNAP to promote expression of rRNA, prevents Rho-dependent transcription termination and regulates correct rRNA folding during ribosome assembly.^71–74^ Studies have shown that the NusB–NusE PPI is the key nucleation point for the formation of the bacterial rRNA transcription complex, rendering this interaction an interesting target for novel antibiotics.^75^ Structural investigation of the PPI interface (∼1600 Å^2^) revealed that the α1-helix and the β2-strand of NusE interact with the binding surface of NusB, formed by two helical bundles, via multiple hydrophobic and hydrophilic contacts (Fig. 6A).^76–79^

Cossar *et al.* used a pharmacophore model based on the NusE α1-helix for a virtual screening of the mini-Maybridge 53 000 compound library and five synthetically accessible hit compounds were further evaluated.^79^ Of these five, compounds **18** and **19** (Fig. 6B) showed inhibition of the *B. subtilis* NusB/NusE PPI in an ELISA assay with IC_50_ values of 6.1 μM and 19.8 μM, respectively. Pyrimidine **18** also displayed moderate inhibition of the growth of Gram-positive *B. subtilis* (9%) and Gram-negative *E. coli* (21%) at a concentration of 200 μM.^79^ In a follow-up study, the same group synthesized four focused compound libraries based on bis-ether **19** and 34 new derivatives were evaluated as NusB–NusE PPI inhibitors, leading to the identification of *cis*-butene iminoguanidine derivative **20** (Fig. 6B) as the most promising compound.^80^ Compound **20** showed good inhibition of the NusB–NusE interaction (55% at 25 μM) and potent antibacterial activity against the Gram-positive pathogens methicillin-resistant *S. aureus* (MRSA) and *S*. *pneumoniae* (MIC ≤ 7 μM), as well as promising activity against the difficult to target, Gram-negative strains *P. aeruginosa* and *Acinetobacter baumannii* (MIC ≤ 125 μM). However, **20** also exhibited considerable cytotoxicity in different human cancer cell lines.^80^

Yang *et al.* used a novel pharmacophore model to perform an *in silico* screen of a combination of the mini-Maybridge compound library and the Enamine antibacterial library.^81^ Seven hit compounds were further evaluated and diarylamine **21** (Fig. 6B) was found to be a potent inhibitor of the NusB–NusE PPI with an IC_50_ of 35 μM. The antibacterial activity of **21** was tested against a panel of representative pathogen strains (*Enterococcus faecalis*, *Klebsiella pneumonia*, *A. baumannii*, *P. aeruginosa*, *Enterobacter cloacae*, *E. coli*, *Proteus vulgaris* and *S. aureus*) and preferential activity of **21** against *S. aureus* was found with a MIC of 60 μM for healthcare-acquired MRSA ST239. Interestingly, **21** displayed no cytotoxicity in mammalian cell lines.^81^

Recently, Qiu *et al.* published two SAR studies based on **21**. In the first study, 60 diarylimine and amine derivatives were synthesized by modifying the left or right benzene ring of **21**.^82^ All new compounds were tested for their antimicrobial activity against a panel of WHO priority pathogens and a series of compounds was tested against further Gram-positive bacteria. Several compounds showed promising antibacterial activities against Gram-negative *A. Baumannii* (lowest MIC 64 μM) and Gram-positive strains *S. pneumonia* (lowest MIC around 27 μM), *S. epidermidis* (MIC ∼ 3 μM), *Staphylococcus saprophyticus* (MIC ∼ 3 μM), *S. pyogenes* (MIC ∼ 27 μM) and *S. agalactiae* (MIC ∼ 27 μM), but none of the compounds were active against Gram-negative *P. aeruginosa* and *E. cloacae*. Selected compounds were also tested against 14 globally spread healthcare-acquired and community-acquired MRSA strains, with some showing stable antibacterial activity with MICs down to around 7 μM. However, it should be noted that the highly active compounds containing an amine linker displayed mild cytotoxicity in human cancer cell lines.^82^ In a second study, a further 38 derivatives of **21**, now named nusbiarylins, with more diverse modifications of the left benzene ring and the linker were prepared.^83^ The new compounds were tested against a panel of clinically significant bacterial strains and some promising antibacterial activities against Gram-positive pathogens were observed, with MIC values as low as 3 μM against *S. saprophyticus* and 13 μM against *S. epidermidis*. As in the previous study, selected compounds were also tested against 14 MRSA strains and consistent antibacterial activities could be observed with MICs down to 7–15 μM making the general class of nusbiarylins promising lead compounds for the development of transcription-targeting anti-MRSA agents.^83^ Very recently, **21** and some of its derivatives were also shown to attenuate the release of staphylococcal virulence factors such as the exoproteins α-toxin and panton-valentine leucocidin *in vitro*, thereby protecting red blood cells from lysis and injury.^84^

### 2.3 Targeting outer membrane protein complexes

Gram-negative bacteria have an outer membrane (OM) that acts as a permeability barrier into the periplasm and excludes many current antibiotics.^85^ Crucial to the barrier function is a network of β-barrel outer membrane proteins (OMPs).^86^ The OMPs are transported to the OM by three essential pathways: lipopolysaccharide transport (Lpt), β-barrel assembly machine (Bam), and the localization of lipoproteins (Lol).^87^ Each of these pathways is comprised of protein complexes and their function is necessary for cell viability. There are currently no antibiotics in clinical use targeting these pathways, so disrupting the PPIs of these complexes is a potential strategy for development of new antibacterial drugs.^88,89^

#### 2.3.i BamA–BamD disruption

The BAM complex is made up of five proteins BamA–E.^90^ BamA is a β-barrel protein which also contains a large soluble domain composed of five periplasmic polypeptide transport-associated (POTRA) domains.^90–94^ The POTRA domains bind to the four lipoproteins BamB–E. BamA and BamD are the only components essential for cell viability and are conserved in all Gram-negative bacteria, making them attractive targets for antimicrobials.^95,96^ A number of compounds have been proposed to target BamA or BamD, resulting in OM structure disruption.^97–103^ While the mechanism of action of certain molecules is not clear, two in particular have been identified as targeting the PPI BamA–BamD.

**Fig. 7:**
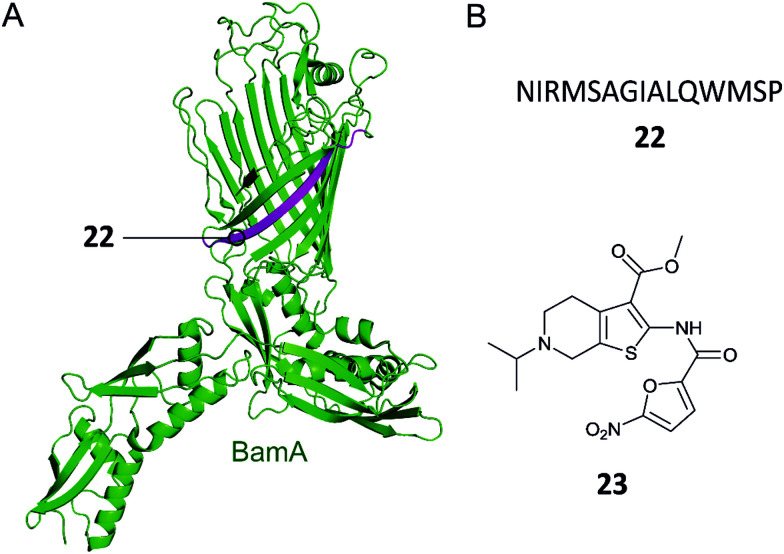
(A) Crystal structure of the *E. coli* BamA with the region mimicked by peptide **22** highlighted in magenta (PDB: 5D0Q).^91^ (B) Peptide and small molecule inhibitors of the BamA–BamD complex.

Hagan *et al.* reported a peptide that inhibits OMP folding by targeting the BamA–BamD interaction in *E. coli*.^101^ Initially, they found that BamD binds to unfolded BamA at the C-terminal of its β-barrel.^101,104^ The authors then used an *in vitro* assay involving the reconstitution of BamA by the BAM complex in proteoliposomes to identify the effect of this interaction on the folding of BamA. It was found that the introduction of a peptide comprised of residues 765–779 of the C-terminus of BamA (**22**, Fig. 7A and B) inhibited the folding of BamA.^101^ Using photo-cross-linking the peptide was confirmed to bind to BamD *in vivo*.^101^ They concluded that peptide **22** mimics the interaction of BamA to BamD, effectively inhibiting the BamA–BamD PPI. Peptide **22** was also found to be toxic when expressed in the *E. coli* periplasm, causing growth defects on plates.^101^ By increasing the permeability of the OM, the sensitivity of the bacteria to antibiotics that cannot normally cross the membrane is enhanced. These promising results demonstrate that peptide **22** is a good starting point for the development of peptidomimetics targeting BamA–BamD that interfere with OMP assembly.

In a recent study, the Si group screened for small molecule compounds disrupting the BamA–BamD PPI in *E. coli* using a yeast two-hybrid (Y2H) screening system.^102^ Of the 25 000 screened compounds, IMB-H4 (**23**, Fig. 7B) was identified as the most specific inhibitor of PPI, where a biolayer interferometry assay identified it as a BamA binder. Further experiments demonstrated that treatment of *E. coli* cells with **23** led to significant damage of the OM. Importantly, **23** was shown to inhibit the growth of *E. coli* ATCC 25922 strain (MIC 10 μM) and other drug-resistant Gram-negative bacteria strains (MIC range 10–80 μM).

#### 2.3.ii Lpt complex disruption

The Lpt macromolecular complex spans the whole cell envelope and is made up of seven proteins, LptA–G.^105,106^ The crystal structure of each component of the complex has been solved, enabling the postulation of an intricate transport mechanism.^107–112^ A lipopolysaccharide (LPS) molecule is extracted by the ATP-binding cassette transporter LptB_2_FG which is bound to the membrane-anchored LptC.^113^ The LPS is transferred to LptC and then across the LptA_2_ bridge in an ATP-dependent manner.^114,115^ LptA_2_ sits in the periplasm and is bound to LptC in the inner membrane and LptD–LptE complex in the OM.^105^ Finally, the LptD–LptE complex translocates the LPS into the outer layer of the OM.^110,116,117^ The N-termini of LptD, LptA and LptC all have a conserved β-jellyroll domain^118^ in the periplasm and associate via PPIs.

**Fig. 8:**
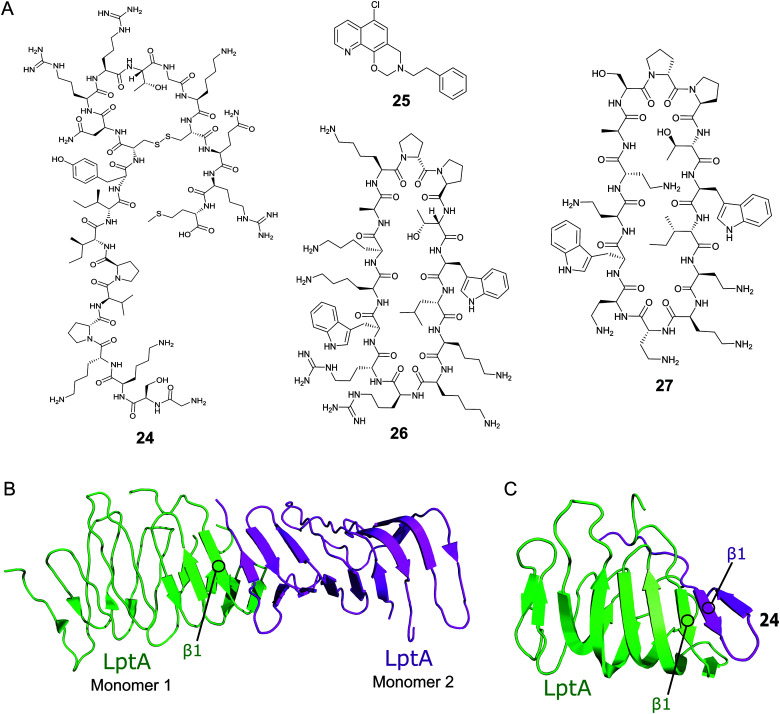
(A) Compounds targeting PPIs of the Lpt complex. (B) Crystal structure of the *E. coli* homodimer LptA complex (PDB: 2R1A).^107^ (C) NMR solution structure of the *E. coli* LptA bound to compound **24**, the N-terminal β1 strand of LptA binds to the N-terminal β1 strand of **24** in a similar manner to the LptA–LptA binding interface (PDB: 6GD5).^120^

Thanatin (**24**, Fig. 8A) is a natural product first isolated in 1996 from the hemipteran insect *Podisus maculiventris* (spined soldier bug).^119^ Antimicrobial activity against several Gram-negative bacteria was reported with strong MICs (<1.5 μM).^119^**24** has since been identified to target LptA and LptD in *E. coli* using photolabeling and MS-based proteomic analysis.^120^ Further studies using a bacterial-two-hybrid screening assay were used to confirm that **24** also inhibits the LptC–LptA interaction in a dose-dependent manner (MIC 0.7–1.4 μM).^121^*In vitro* FP studies identified **24** as binding to LptA and LptD with nanomolar affinities and SPR and NMR measurements confirmed that **24** disrupts the LptC–LptA interaction.^120,121^ The NMR solution structure of LptA–**24** complex was solved, where **24** was found to interact with the first N-terminal β-strand in the β-jellyroll of LptA (Fig. 8C).^120^ The crystal structure of LptA_2_ shows how the LptA subunits interact head-to-tail through their N- and C-β-strands (Fig. 8B).^107^ The binding site of **24** overlaps with the LptA–LptA binding interface, therefore blocking the dimerization of the LptA monomers.^120^ Both the N-terminal β-strands of LptD and the C-terminal β-strands of LptC share similarities with LptA, where residues involved in LptA–**24** binding were found to be highly conserved.^108^

The Si group used a Y2H screening system in *E. coli* to identify small molecule IMB-881 (**25**, Fig. 2A) targeting LptA–LptC.^122^ Using SPR, **25** was found to disrupt the LptA–LptC interaction by specifically binding to LptA.^122^ Electron microscopy demonstrated that treatment of *E. coli* cells with **25** led to significant damage of the OM, and antibody detection indicated a higher level of LPS in the periplasm, suggesting **25** inhibits its transport *in vivo*. **25** was then found to greatly inhibit the growth of *E. coli* ATCC 25922 strain (MIC of 19 μM), and other clinical multidrug-resistant strains (MIC range of 19–150 μM).

A macrocyclic β-hairpin peptidomimetic, L27-11 (**26**, Fig. 8A), was initially optimised via a number iterative peptidomimetic library synthesis cycles.^123,124^ Further optimisation led to murepavadin (**27**, Fig. 8A), a clinical candidate with an MIC of 5.15 nM against >1000 clinical multi-drug resistant *P. aeruginosa* strains.^124,125^ It reached stage three of clinical trials, but trials are currently suspended due to nephrotoxicity.^88^ Photoaffinity experiments identified LptD as the target for this new family of compounds and mechanistic studies confirmed they inhibit LPS transport.^124,126^ Photolabeling suggested that the peptidomimetics bind the periplasmic domain of LptD and point mutations indicate that the point of interaction is close to the β-jellyroll domain.^127,128^ This suggests that the compounds may bind to LptD at the LptD–LptA binding interface and inhibit the PPI.

### 2.4 Targeting toxin–antitoxin systems

The toxin–antitoxin (TA) systems are present in a large variety in bacteria cells.^129^ There are many TA families broadly categorised into six types (I–VI) based on the inhibition of toxin function by the antitoxin.^130,131^ The function of toxins is not fully understood, they were originally identified as conferring plasmid stabilisation and phage resistance.^132,133^ Since then a number of functions for TA systems have been proposed, such as programmed cell death, biofilm formation,^134^ regulation of virulence factors in pathogenic bacteria,^130^ persistence and dormancy.^135^ The TA systems are ubiquitously present in bacterial genomes providing a class of interactions that can be targeted in multiple bacteria systems.^136^

Type II TA systems are the most abundant in bacteria and the most well characterised.^131^ They are comprised of a proteic toxin and a proteic antitoxin where the antitoxin directly binds to and inhibits the toxin. The type II toxins can exert toxicity in several ways including inhibition of translation or replication and interruption of cell wall synthesis, although the majority affect translation. In normal growth conditions, the stable toxin is prevented from exerting its lethal effect through tight binding with its less stable antitoxin partner. The antitoxin also regulates expression of the type II TA operon by binding to its operator site.^133^

Type II TA systems have, in recent years, gained much interest as targets for antibiotic agents as well as to counter persistence.^137^ A number of reviews have identified TAs as a possible target for new antibiotics with the aim to activate the toxin, essentially allowing bacterial suicide.^136,138–142^ The toxin can be liberated by targeting the TA PPI via either (a) competitive binding to the antitoxin or (b) dissociation of the existing TA complex. Additionally, identifying molecules that bind to the toxin of a TA system, either mimicking the antitoxin binding at the PPI interface or targeting an allosteric position, is a potential strategy for combatting bacterial persistence.^131,143^

In this section, recent studies involving the rational design and high throughput screening of peptides and small molecule compounds to perturbate TA PPI interfaces will be summarised. For clarity, the TA systems have been categorised by the targeted cellular function and structural class.

#### 2.4.i TA pairs targeting translation

Most toxins in bacteria have been found to target translation, with specific targets being mRNA, tRNA or translation machinery such as ribosomes.^144^ Antibacterial agents targeting toxins carrying the classical folds VapB, PemK, HicB and HipB have been identified. In all these cases a simple starting point has been identified but no molecules have, as yet, progressed into clinical research.

##### 2.4.i.1 VapBC

In a comprehensive systematic genomic analysis of the TA genome in *M. tuberculosis*, 47 of 88 putative TA systems were identified to belong to the VapBC family.^145^ The VapC toxins have a characteristic PIN (PilT N-terminus) domain in which four acidic amino acid residues are conserved. The conserved residues are crucial for ribonuclease activity, and the VapC toxins inhibit translation via mRNA cleavage. Each VapB antitoxin inhibits the ribonuclease activity of its cognate toxin. The VapBC family is an attractive target for novel antibacterial therapies since liberating the toxin could lead to cell death due to hindered translation. Structural information for VapBC3, VapBC5, VapBC11, VapBC15, VapBC26 and VapBC30 has been acquired and efforts to rationally design peptides to disrupt the TA complexes have been carried out.^146^

**Fig. 9:**
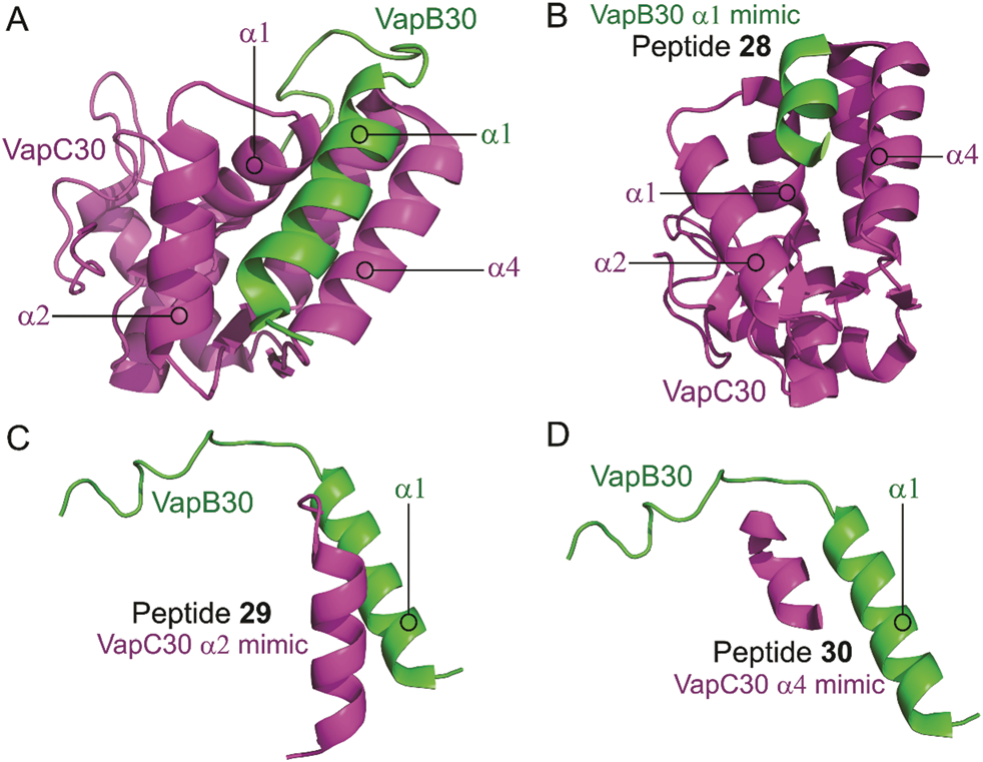
(A) Crystal structure (PDB: 4XGQ)^147^ of the complex between VapB30 (green) and VapC30 (magenta). A representation of the regions selected for the design of peptides **28** (VapB30 helix α1 mimic), **29** (VapC30 helix α2 mimic) and **30** (VapC30 helix α4 mimic) is shown (B), (C) and (D), respectively.

Lee *et al.* characterised the structures of the *M. tuberculosis* VapBC30 in 2015 and VapBC26 in 2017 and identified the key residues involved in binding at the TA interface.^147,148^ They then rationally designed peptides to mimic the binding interface and interfere with the TA PPI thereby activating the ribonuclease activity of the VapC toxin. The VapBC30 complex comprises three structural components: α1-helix of VapB30 (residues 49–62), α2-helix of VapC30 (residues 17–27), and α4-helix of VapC30 (Fig. 9).^147^ The authors rationally designed three peptides to mimic each of these components to inhibit TA binding of VapBC30. Peptide **28** mimics the α1-helix of VapB30, designed to compete with VapB30 for interaction with VapC30 (Table 2). Peptides **29** and **30** mimic the α2- and α4-helical regions of VapC30 respectively, designed to compete with VapC30 for interaction with VapB30 (Table 2 and Fig. 9). The ability of the peptides to disrupt the TA complex was determined with a fluorescence reporter assay measuring the ribonuclease activity of VapC30. The inhibitory potency was calculated considering the relative ribonuclease activity, where the toxin alone (10 μM) has a 0% inhibitory value and the TA complex (10 μM) represents 100% inhibition. The peptides were able to disrupt the TA complex in a concentration dependent manner (10–100 μM). At the highest concentration (protein to peptide 1 : 10) the VapC30 toxin-mimicking peptides **29** and **30** showed a greater inhibitory effect of 53% and 73% compared to the antitoxin-mimicking peptide **28** at 43%.

**Table 2:** Peptides targeting the VapBC30 and VapBC26 TA systems

TA complex	Mimicked protein	Mimicked region	Peptide (number in reference)	Peptide sequence (residues)	Peptide concentration (μM)	% inhibition of PPI
*VapBC30* ^147^	VapB30	α1	**28** (I)	ELAAIRHR (52–59)	100	43^b^
	VapC30	α2	**29** (I)	DEPDAERFEAAVEADHI (14–30)	100	53^b^
	VapC30	α4	**30** (III)	RPGEPGGRE (48–56)	100	73^b^

*VapBC26* ^148^	VapB26	Coil between α2 and α3	**31** (I)	PPPRGGLYAGSEPIA (44–58)	—	—
	VapB26	α3	**32** (II)	VDELLAGF (61–68)	—	—
	VapC26	α1	**33** (III)	ALLAYFDAAEP (7–17)	NR	NR^a^
	VapC26	α3	**34** (IV)	PYVVAELDYLVATRVG (37–52)	NR	NR
	VapC26	α4	**35** (V)	DAELAVLRELAG (54–65)	200	80^c^
	VapC26	Partial α3 and α4	**36** (VI)	YLVATRVGVDAELAV (45–59)	NR	NR
	VapC26	Whole α3 and α4	**37** (VII)	PYVVAELDYLVATRVGVDAELAVLRELAG (37–65)	NR	NR

a NR: not reported.

b Toxin and TA complex concentration 10 μM.

c Toxin and TA complex concentration 2.5 μM.

**Fig. 10:**
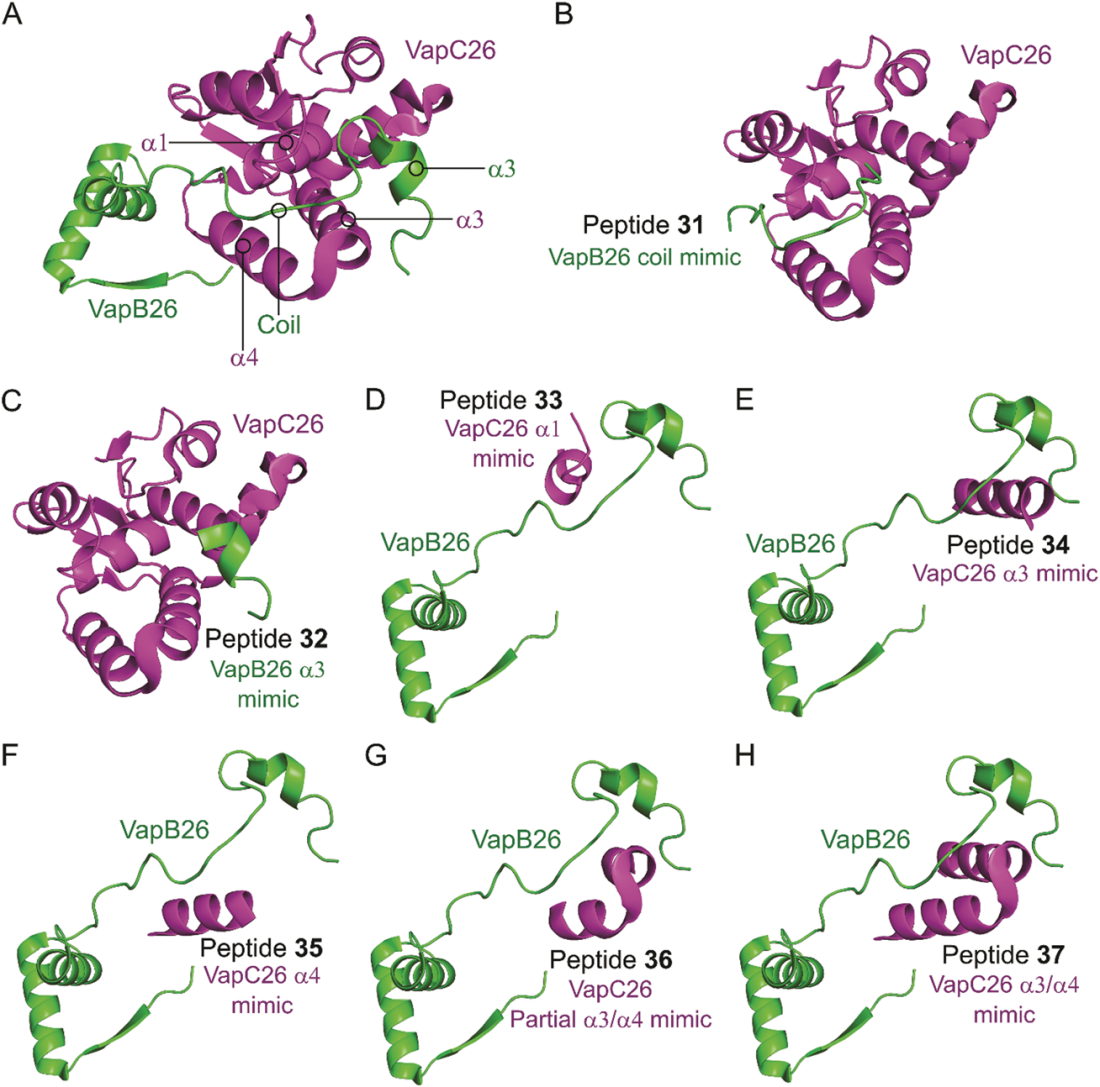
(A) Crystal structure (PDB: 5X3T)^148^ of the complex between VapB26 (green) and VapC26 (magenta). A representation of the regions selected for the design of peptides **31** (VapB26 coil mimic), **32** (VapB26 helix α3 mimic), **33** (VapC26 α1 mimic), **34** (VapC26 α3 mimic), **35** (VapC26 α4 mimic), **36** (VapC26 partial α3/α4 mimic) and **37** (VapC26 α3/α4 mimic) is shown in (B), (C), (D), (E), (F), (G) and (H), respectively.

In the VapBC26 complex, the active site of VapC26, composed of α1-, α3- and α4-helices, interacts with the α3-helix and the C-terminal region of VapB26.^148^ The authors designed seven peptides (**31–37**) to disrupt the TA pair, based on the binding interface (Table 2 and Fig. 10). The inhibitory potency was calculated considering the relative ribonuclease activity, where the toxin alone (2.5 μM) has a 0% inhibitory value and the TA complex (2.5 μM) represents 100% inhibition. Peptides **31** and **32** mimicking the VapB26 antitoxin did not show any effect on enzymatic activity. The remaining five peptides were designed to mimic the VapC26 toxin to compete with VapC26 for interaction with VapB26. Peptides **34** and **35**, mimicking the α3- and α4-helices of the VapC26 toxin, respectively, and peptide **37**, composed of both the α3- and α4-helices increased the ribonuclease activity in a concentration dependent manner (2.5–1 mM). Peptide **35** demonstrated the best ribonuclease activity and was able to disrupt TA binding of VapBC26 by 80% at 200 μM (protein to peptide 1 : 80).

**Fig. 11:**
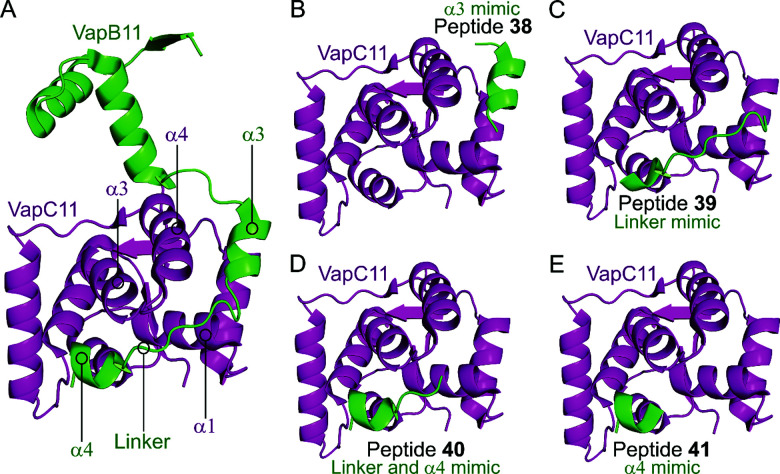
(A) Crystal structure (6A7V)^149^ of the complex between VapB11 (green) and VapC11 (magenta). A representation of the regions selected for the design of VapB11 peptides **38** (helix α3 mimic), **39** (linker mimic), **40** (linker and helix α4 mimic) and **41** (helix α4 mimic) is shown in (B), (C), (D) and (E), respectively.

Deep *et al.* carried out a similar experiment, characterising the VapBC11 TA complex of *M. tuberculosis*, and designed peptide inhibitors of the interface.^149^ The authors in this case were trying to identify peptides that could inhibit ribonuclease activity. Considering bacterial persistence and tolerance, they postulated that the inactivation of VapC11 could allow a bacterial cell to be more susceptible to clearance. Based on a crystal structure, it was found that the TA interface covers a large area of 1375 Å^2^. The C-terminal residues of VapB11 wrap around a VapC11 cavity formed by its α1-, α3- and α4-helices. Four overlapping peptides (**38–41**, 10–14 residues long) mimicking the VapB11 antitoxin were designed (Table 3 and Fig. 11) and the change in ribonuclease activity was measured using *in vitro* tRNA-Leu^CAG^ cleavage assays. All four peptides inhibited the activity of VapC11 to some degree, the inhibition was dependent on a 100-fold greater amount of peptide compared to protein.

**Table 3:** Peptide inhibitors of the VapBC11 TA system

TA complex	Mimicked protein	Mimicked region	Peptide (number in reference)	Peptide sequence (residues)
*VapBC11* ^149^	VapB11	α3	**38** (I)	LSREFLLGLE (41–50)
	VapB11	α3 and flexible linker	**39** (II)	EGVGWEGDLDD (50–60)
	VapB11	Flexible linker and α4	**40** (III)	WEGDLDDLRSDRPD (54–67)
	VapB11	α4	**41** (IV)	LDDLRSDRPD (58–67)

In a recent study, Sundar *et al.* designed peptide inhibitors **42–47** mimicking the published toxin structures of VapBC3, VapBC5, VapBC11, VapBC15, VapBC26 and VapBC30 using the Peptiderive server (Table 4).^150^ The protein–peptide docking was performed using the Cluspro server to identify the binding affinity of the peptides to the toxins. Peptides with a strong interface score were found for each target. Interestingly, the peptides for VapBC26 and VapBC30 reported here have lower binding energy compared to the ones previously identified, however, this has only been measured *in silico* and further biochemical characterisation is necessary to confirm binding affinities.^147,148,150^

**Table 4:** Toxin-derived peptide inhibitors of VapBC TA systems

TA complex	Mimicked protein	Peptide	Peptide sequence
*VapBC3* ^150^	VapC3	**42**	VTAADLRRLR
*VapBC5* ^150^	VapC5	**43**	RGAQADPGLR
*VapBC1* ^150^	VapC1	**44**	FLLGLEGVGW
*VapBC15* ^150^	VapC15	**45**	ALALQGSGFD
*VapBC26* ^150^	VapC26	**46**	MYAGSEPIAR
*VapBC30* ^150^	VapC30	**47**	ELAAIRHRCA

##### 2.4.i.2 HicBA

**Fig. 12:**
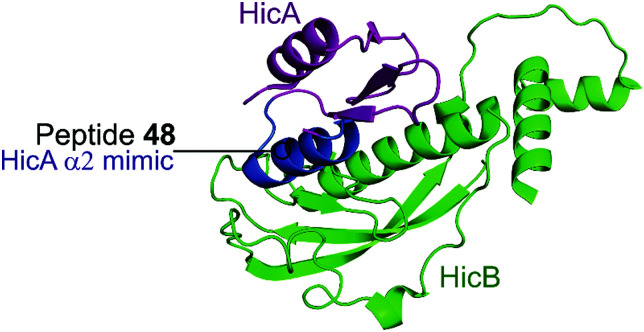
Crystal structure (PDB: 5YRZ)^152^ of the complex between HicA (magenta) and HicB (green) from *S. pneumoniae*. The region of HicA mimicked by peptide **48** is highlighted in blue.

HicBA is a newly identified, relatively uncharacterised type II TA system.^151^ The Lee group characterised the structure of HicBA from *S. pneumonia* and designed peptides to target the TA interface in a similar manner to their studies of VapBC in *M. tuberculosis.*^147,148,152^ The ribonuclease activity of HicA toxins relies on a RNA binding domain, which contains a conserved histidine residue (H36 in *S. pneumoniae).*^152,153^ The N-terminal region of the HicB antitoxin of *S. pneumoniae* sterically blocks the toxin active site, thereby covering 1183 Å^2^ of the toxin. The earlier studies targeting VapBC26 and VapBC30 suggested toxin mimicking peptides had the greatest inhibitory effect of the interaction. The authors therefore designed four peptides (**48–51**) mimicking the α2-helix of HicA (Table 5 and Fig. 12), the inhibitory potency was calculated considering the relative ribonuclease activity, where the toxin alone (4 μM) has a 0% inhibitory value and the TA complex (4 μM) represents 100% inhibition. Peptide **48** demonstrated a concentration dependent inhibition of the interaction and increased ribonuclease activity (2–16 μM), 80% at the highest concentration (protein to peptide 1 : 4). Peptide **48** also demonstrated potent antibacterial activity against the Gram-positive pathogens *B. subtilis*, *S. aureus* and *S. epidermis* (MIC 6–12.5 μM), as well as promising activity against the difficult to target, Gram-negative strain *P. aeruginosa* (MIC 6 μM). Peptide **48** contains 11 residues of the full α2-helix, including seven residues that interact with HicB, and possesses the highest helicity out of the four peptides tested.

**Table 5:** Peptide inhibitor of the HicBA TA system

TA complex	Mimicked protein	Mimicked region	Peptide	Peptide sequence (residues)	Peptide concentration (μM)	% inhibition of PPI
*HicBA* ^152^	HicA	α2	**48**	ELNKYTERGIRKQAG (53–67)	16	80^b^
	HicA	α2	**49**	GELNKYTERGIRKQAG (52–67)	NR	NR^a^
	HicA	α2	**50**	ELNKYTERGIRKQAGL (53–68)	NR	NR
	HicA	α2	**51**	GELNKYTERGIRKQAGL (52–68)	NR	NR

a NR: not reported.

b Toxin and TA complex concentration 4 μM.

##### 2.4.i.3 PemK-like protein family

The PemK-like protein family includes a number of homologs which have been identified in most bacterial systems including the MazF toxin in *E. coli*, *P. aeruginosa*, *S. aureus*, *B. subtilis* and *M. tuberculosis* and the MoxT toxin in *Bacillus anthracis*.^154^ The toxins share a ribonuclease SH3-like barrel fold domain and an antitoxin with a RHH motif at the N-terminus. Three or four α-helices link the two β-sheets of the toxin monomer. The MazEF toxin–antitoxin pair is very well characterised in pathogenic bacteria.^155^ The MazF toxin superfamily members are proposed to have a conserved mode of binding to their antitoxin using two binding pockets located on the β1–β2 and β3–β4 linkers. The disordered C-terminus of the antitoxin binds both via a hydrophobic group to site 1 and via an extensive interface with the less conserved site 2.^156^ Site 2 is the known ribonuclease site, but disruption of either pocket is predicted to affect the ribonuclease activity.

**Fig. 13:**
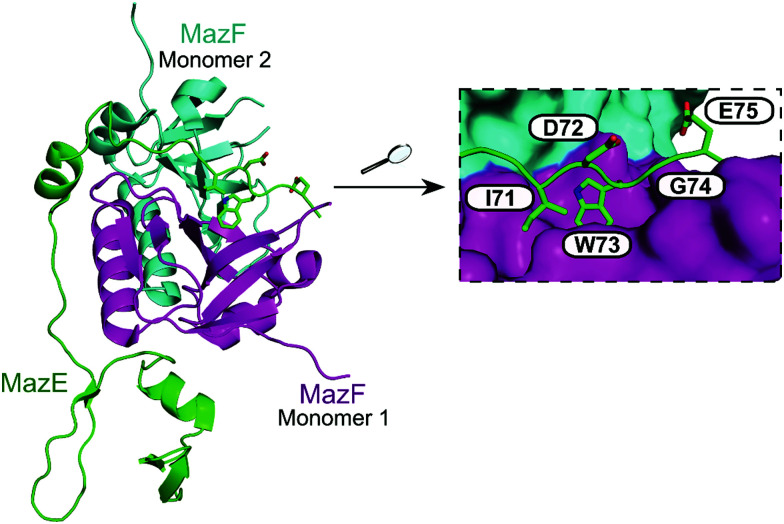
Crystal structure of the *E. coli* MazEF TA complex (PDB: 1UB4)^160^ with MazF toxin monomers shown in magenta and cyan, and MazE antitoxin in green. Enlarged picture (right chart) highlights MazE residues 71–75 (IDWGE, in green) that are crucial for the interaction with MazF (magenta and cyan surfaces represent, respectively, monomers 1 and 2).

The MazEF pair has been implicated in the programmed cell death (PCD) of *E. coli* bacteria as a suicide module under stress conditions via activation of the toxin.^157^ This cell death requires activation by a linear peptide termed the extracellular death factor (EDF), a quorum sensing molecule.^158^ Using a continuous fluorescence reporter assay the authors found that the EDF amplified the endoribonucleolytic activity of the MazF toxin (0.25 μM) in a concentration dependent manner (1.5–7.5 μM), up to 57% increased activity.^159^ Additionally, the EDF prevented the inhibitory effect of the antitoxin MazE, and in the presence of MazE (0.025 μM) and increasing concentrations of the EDF (0–15 μM), MazF (0.25 μM) had activity close to 100% (1 : 60 MazF to EDF). The EDF (sequence: NNWNN) was shown by computational modelling to bind to MazF in a similar way to MazE-MazF binding, with parallel contacts between the EDF with MazF and the binding site of MazE 71–75 (IDWGE) (Fig. 13).^159^ It has been suggested that MazE binds to one mRNA binding site of MazF and this initial binding interrupts the other mRNA binding site, resulting in MazF inhibition. The EDF, however, only binds to one binding site and the other site is still available to bind and cleave mRNA thereby allowing MazF activity and preventing inhibition by MazE.

There are seven MazEF homologs in *M. tuberculosis*, of which several are proposed to have the same effect on PCD as in *E. coli.* A linear pentapeptide EDF (sequence: ELWDR) was identified for MazEF4 of *M. tuberculosis*.^161^ Interestingly, the charge distribution of the MazEF4 binding site is opposite to that of the MazEF of *E. coli.* The final positive arginine residue of the EDF reflects this shift and binds to the negative pocket of MazF, otherwise based on NMR interaction studies the EDF binds in a similar manner as in the *E. coli* homolog. This EDF was also identified as enhancing the ribonuclease activity of MazF4 and disrupting the MazEF4 complex.

There is divergence in the EDF sequences in different species or homologs within a species and other EDFs discovered from *B. subtilis* and *P. aeruginosa* were found to enhance activity of MazF in *E. coli*.^162^ However, only the EDF isolated from *E. coli* directly interrupts MazE–MazF binding of *E. coli*.^163^ The EDFs of *E. coli* and *P. aeruginosa* were found to enhance the activity of MazF-mt3 and MazF-mt6 of *M. tuberculosis*, although the effect was mainly observed from the EDF of *E. coli*.^164^ The specific EDF peptides for each MazEF pair are a possible starting point for a new class of antibiotic peptides to target MazEF mediated cell death. Furthermore, the ability of EDFs from other species to enhance the activity of homologs may be a useful starting point to design a peptide capable of targeting multiple organisms.

**Fig. 14:**
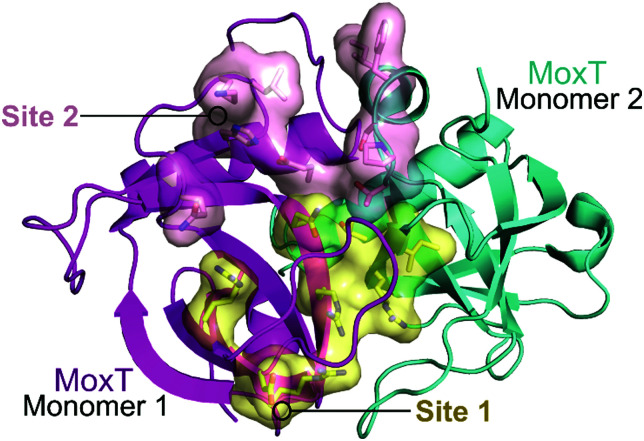
Crystal structure of MoxT dimer (PDB: 4HKE)^165^ with monomers 1 and 2 shown in magenta and cyan, respectively. Interaction sites 1 and 2 are shown as yellow and pink surfaces, respectively.

In 2007, Agarwal *et al.* identified a PemK-like toxin, later termed MoxT, in *B. anthracis.*^166^ In a series of papers the group rationally designed peptides targeting the MoxX antitoxin/MoxT toxin interface, some mimicking EDFs.^165,167,168^ The MoxXT PPI was initially modelled based on *E. coli* MazEF and the C-terminus of the MoxX antitoxin was implicated in toxin binding. In the first study, six peptides (**52–57**, Table 6) mimicking the C-terminus of either the MoxX or MazE antitoxins were designed to disrupt the toxin–antitoxin interaction at sites 1 or 2 (Fig. 14).^167^ ELISA was used to identify the effect on the peptides on the TA interaction, the cells were coated with MoxX (200 ng) and preincubated MoxT (200 ng) with peptide (2 μM) was added. The peptides targeting site 2 demonstrated the greatest inhibition (up to 35%) of the TA interaction compared to those targeting site 1. Peptides **54** and **56** (mimicking site 2 of MoxX) inhibited the interaction most effectively but also inhibited the activity of the toxin. In a second study, following homology modelling of the MoxXT complex, a novel peptide (**58**) was designed to mimic MoxX binding to site 2 (Table 6).^168^ This peptide (2 μM) was able to inhibit the TA interaction by 42%, however it also demonstrated inhibition of MoxT ribonuclease activity. To preserve the ribonuclease activity, site 1 rather than site 2 must be targeted, however in the above studies the peptides designed to mimic site 1 had little effect on the toxin–antitoxin interaction.

**Table 6:** Peptide inhibitors of the MoxXT TA system

TA complex	Mimicked protein	Mimicked region	Peptide (number in reference)	Peptide sequence (residues)	Peptide concentration (μM)	% inhibition of PPI
*MoxXT* ^167^	MoxX	Site 1	**52** (I)	VERLVSGG (88–95)	2	11^a^
	MazE	Site 1	**53** (II)	NLHRNIW (66–73)	2	20^a^
	MoxX	Site 2	**54** (III)	LLFQHLTE (44–51)	2	35^a^
	MoxX	Site 2	**55** (IV)	KRYQHESM (52–59)	2	25^a^
	MoxX	Site 2	**56** (V)	RRGYIEMG (60–67)	2	30^a^
	MazE	Site 2	**57** (VI)	KAELVNDI (55–62)	2	22^a^
*MoxXT* ^168^	MoxX	Site 2	**58**	SKIGAWAS	2	42^a^
*MoxXT* ^165^	*E. coli* EDF	Site 1	**59** (I)	NNWDN (1–5)	1	37^b^
	*E. coli* EDF	Site 1	**60** (II)	NNWNN (1–5)	1	44^b^
	*E. coli* EDF	Site 1	**61** (III)	DNWNN (1–5)	1	22^b^
	*B. anthracis* EDF	Site 1	**62** (IV)	SIWNK	1	40^b^
	MazE	Site 1	**63** (V)	HENIDW (68–73)	1	31^b^
	MazE	Site 1	**64** (VI)	ENIDWGEP (69–76)	1	27^b^
	MoxX	Site 1	**65** (VII)	RGYIEMG (61–67)	1	46^b^

a Toxin and antitoxin 200 ng.

b Toxin 0.23 μM and antitoxin 300 ng.

A third study involved the rational design of peptides to disrupt the TA interaction of MoxXT and importantly aimed to stimulate the activity of MoxT by targeting site 1.^165^ Peptides **60–65** were designed to mimic the antitoxins MoxX and MazE of *E. coli* as well as the EDF of *E. coli* and predicted EDF of *B. anthracis* (Table 6). ELISA was used to identify the effect on the peptides on the TA interaction, the cells were coated with MoxX (300 ng) and preincubated MoxT (0.23 μM) mixed with peptide at increasing concentrations (0.2–1 μM) was added. All inhibited the MoxXT interaction, with maximum efficacy reached at 1 μM, peptides **60** and **65** proved to be most successful with inhibition of 44% and 46%, respectively. A continuous fluorometric assay, whereby fluorophores attached to RNA probes emit fluorescence when cleaved, identified an increase in ribonuclease activity in the presence of 40 equivalents of peptide compared to MoxT. Peptides **59**, **62** and **64** demonstrated the greatest enhancement in the ribonuclease activity of MoxT. The authors proposed that the helical structures reported in the first studies too closely mimic the antitoxin and bind at site 2 rather than site 1. The non-helical (C-shaped or extended structure) peptides with one aromatic residue that are reported in their later study cause an increase in MoxT activity.

Recently, a number of papers have reported *in silico* approaches to the design of peptides and peptidomimetics to interfere with the MazEF interface.^150,169,170^ Sundar *et al.* designed peptide **66** using the Peptiderive server to mimic the EDF of MazF4 of *M. tuberculosis* (Table 7).^150^ Unfortunately the *in silico* experiments using the Cluspro docking server identified the peptide to have worse binding affinity to MazF than its EDF. Using the same Peptiderive and Cluspro server, Mohammadzadeh *et al.* designed peptides **67–69** mimicking MazE of the MazEF TA system in *L. monocytogenes*.^169^ It is not clear whether these peptides bind with a similar affinity to MazF as MazE, but they interact at the same binding site on the toxin.

**Table 7:** Peptide inhibitors of MazEF TA systems

TA complex	Species	Mimicked protein	Peptide	Peptide sequence
*MazEF4* ^150^	*M. tuberculosis*	EDF	**66**	AYPYESEAER
*MazEF* ^169^	*L. monocytogenes*	MazE	**67**	RDEMERGYAE
	*L. monocytogenes*	MazE	**68**	RDEMERGYAEMA
	*L. monocytogenes*	MazE	**69**	MERGYAEMATINFA

Farhadi *et al.* modelled MazF of *A. baumannii* and designed peptidomimetics based on the *E. coli* EDF using the pep:MMs:MIMIC tool.^170^ The compounds were docked with MazF and the nine highest ranking mimetics had low binding energies of less than −6.8 kcal mol^−1^, lower than the original peptide (−5.2 kcal mol^−1^), demonstrating favourable docking. Several mimetics formed two hydrogen bonds with MazF, compared to one formed by the original peptide. Some of the *in silico* designed peptides and peptidomimetics may be a starting point for novel drugs to target MazF and mediate cell death. Considering the results of the Bhatnagar group suggesting that only targeting site 1 results in enhanced MazF activity, biochemical experiments of peptide–protein affinity and importantly their effect on MazF activity must first be considered.

##### 2.4.i.4 HipAB

**Fig. 15:**
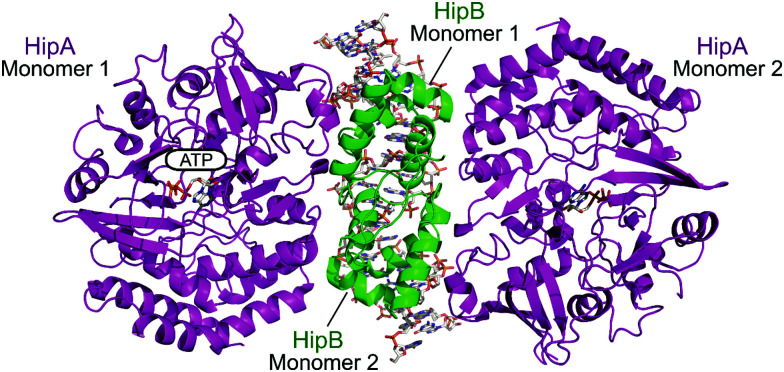
Crystal structure of the *E. coli* HipAB TA complex bound to DNA (PDB: 3HZI)^172^ with HipA toxin monomers shown in magenta and HipB antitoxin monomers shown in green. The DNA and HipA-bound ATP are shown as sticks.

The HipAB TA system was the first TA system to be implicated in persistence in *E. coli* and it has since been identified in a number of pathogenic bacteria.^171^ The structure of *E. coli* HipAB was characterised in 2009 by Schumacher *et al.* (Fig. 15).^172^ The HipA toxin was found to have a serine kinase-like fold and has kinase activity. It was originally suggested to phosphorylate the elongation factor thermo unstable (EF-Tu).^172^ It was later found the target was further upstream in the translation process, it phosphorylates, and therefore inactivates, the glutamyl-tRNA-synthetase (GltX).^173,174^ This leads to inhibition of protein biosynthesis, a process often linked to the initiation of bacterial dormancy and persistence.^173,174^ In the presence of the HipB antitoxin its activity is neutralised; the HipA N-terminal domain interacts with one HipB subunit whilst the C-terminal domain interacts with the other HipB subunit. Strangely the HipB monomers do not directly interact or occlude the HipA active site, suggesting that HipB simply locks HipA into an open inactive conformation that prevents binding of protein substrates.

**Fig. 16:**
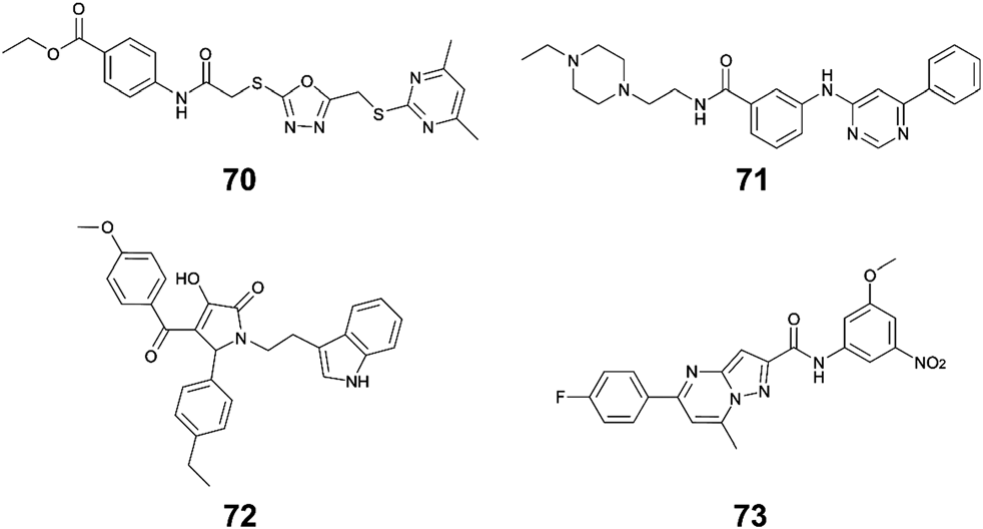
Small molecule inhibitors of HipA toxin.

Li *et al.* used *in silico* structure based computational screening followed by *in vitro* experiments to identify novel small molecules inhibiting HipA, thereby preventing persistence.^175^ The Chemdiv kinase and SPECS compounds libraries were used together with docking with Glide to identify tight binders. SPR was used to measure hit HipA binding affinity, and the tightest binders were tested for their effect on persistence. The number of colonies present following addition of hit molecules before and after antibiotic treatment were compared to quantify the persister fraction. 14 molecules reduced *E. coli* persistence at 250 μM and did not cause cytotoxicity. The EC_50_ values of the four most potent compounds (**70–73**, Fig. 16) were found to be less than 126 μM, with compound **72** having the highest anti-persister activity (46 μM, Table 8). The *K*_d_ values also demonstrated **72** as the tightest binder (270 nM). These molecules were found to target the active site of the HipA toxins, and bind in a similar manner to ATP, rather than targeting the PPI interface. A similar methodology, perhaps screening for peptides targeting the HipAB interface, would be a strategy to identify other molecules that could target HipA mediated persistence.

**Table 8:** Activities of small molecule inhibitors of HipAB TA system

TA complex	Species	Compound (number in reference)	*K*_d_ (μM)	EC_50_ (μM)
*HipAB* ^175^	*E. coli*	**70** (1)	54	126
	*E. coli*	**71** (2)	43	84
	*E. coli*	**72** (3)	0.27	46
	*E. coli*	**73** (4)	35	116

#### 2.4.ii TA pairs targeting cell wall synthesis

##### 2.4.ii.1 ε_2_ζ_2_/PezAT

The zeta toxin–epsilon antitoxin complex (ε_2_ζ_2_) is a TA system often found in multidrug-resistant bacteria pathogens and is also present in *S. pneumoniae* or *Streptococcus suis* as PezAT.^139,176^ The ζ/PezT toxin is a kinase with an ATP/GTP binding site and a core fold of phophotransferases which phosphorylates and inactivates uridine diphosphate-*N*-acetylglucosamine (UNAG) amino sugars, preventing their involvement in cell wall peptidoglycan biosynthesis.^177^ In stable cell conditions the complex is found in a heterotetrameric form, and the ζ toxin is neutralised by the ε antitoxin. In absence of the antitoxin, the toxin induces reversible proliferation of cells in a population due to inhibited peptidoglycan synthesis.^178^ A prolonged disruption of the complex leads to irreversible proliferation by cell wall autolysis.

The crystal structure of the *S. pyogenes* ε–ζ system was characterised in 2003.^179^ The ε_2_ζ_2_ binding interface involves the α-helix of ε binding to a groove of the ζ toxin composed of three α-helices. Lioy *et al.* used a cell-based HTS assay using BRET technology to identify a disruptor of the *S. pyogenes* ε–ζ PPI.^180^ They used the crystal structure to carry out molecular dynamic studies to define the main interacting residues of the PPI. They theorised that a compound mimicking the toxin residues that interacts with the antitoxin would free the toxin to trigger growth inhibition. *In silico* modelling of peptide profiles was used to direct the choice for an extensive collection of peptide libraries that were then used to screen for hits targeting the interaction. They fused the N-terminus of ε and C-terminus of ζ with reporter genes and tested for a decrease in BRET signal relative to controls to identify hit compounds interrupting the ε–ζ interaction. Two of 17-residue peptide sub-libraries resulted in a decrease in BRET signal which was lost upon sub-fractionation of the libraries. Therefore, the authors proposed that the disruption was a result of more than one binder, that when combined can disrupt the PPI. Their experiment validated ε_2_ζ_2_ as a potential target, proving it can be disrupted with oligopeptides. It is not clear whether ε or ζ is targeted and if the residues involved in the PPI are the same as those involved in peptide binding. It is possible that the peptides block ATP binding rather than restoring kinase activity of the toxin.

**Fig. 17:**
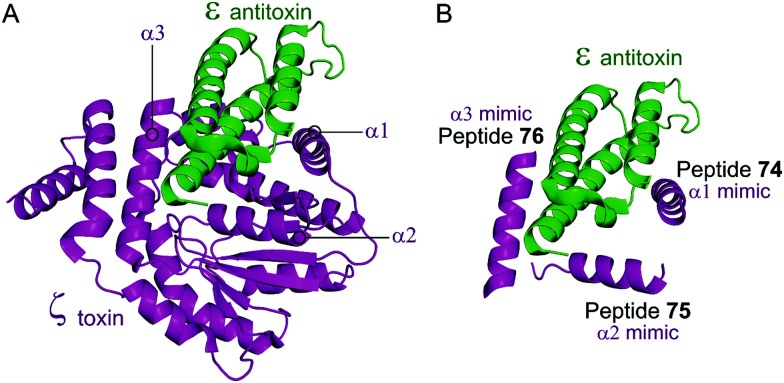
(A) Binding interface between the ε antitoxin (green) and the ζ toxin (magenta) observed in the crystal structure of the tetrameric ε_2_ζ_2_ complex (PDB: 1GVN).^179^ (B) Representation of the regions selected for the design of ζ-derived peptides **74** (helix α1 mimic), **75** (helix α2 mimic) and **76** (helix α3 mimic).

Based on the crystal structure from *S. pyogenes*, and validation that the TA pair can be disrupted, Fernández-Bachiller *et al.* rationally designed peptides to target the ε–ζ PPI.^181^ They initially designed three peptides **74–76** based on the three α-helices of the ζ toxin to target ε (Table 9 and Fig. 17). Due to their high propensity for aggregation, several analogues of **74–76** were designed, possessing shorter sequences and amino acid substitutions in order to improve aqueous solubility. The binding affinity of each peptide to the ε antitoxin was determined by FP. Peptide **77** (Table 9 and Fig. 17), a shorter analogue of **73** where Leu20 was replaced by a His residue, was found to have the tightest binding (*K*_d_ = 75 nM) amongst all peptides. This substantial improvement when compared to the affinity of the ε–ζ complex (*K*_d_ = 1 μM) making peptide **77** a promising starting point for the design of an antibacterial agent that can trigger ζ-toxin mediated autolysis.

**Table 9:** Peptide inhibitors of the ε_2_ζ_2_ TA system

TA complex	Mimicked protein	Mimicked region	Peptide (number in reference)	Peptide sequence (residues if applicable)	*K*_d_ (nM)
*ε*_*2*_ *ζ*_*2*_^181^	ζ	α1	**74** (I)	TDKQFENRLNDNLEELIQ (8–25)	—
	ζ	α2	**75** (II)	GSGKTSLRSAIFEETQ (43–58)	—
	ζ	α3	**76** (III)	INSYLGTIERYETMYADD (149–165)	—
	ζ	α1	**77** (I_a_)	LNDNHEELIQ	74.5

#### 2.4.iii TA pairs with lipase activity

##### 2.4.iii.1 TplE–TplEi

TplE is a phospholipase family protein belonging to the Tle4 ligase family. Its antibacterial lipolytic activity is neutralised by the TlpEi antitoxin.^182^ The crystal structure of *P. aeruginosa* TplE–TlpEi has recently been characterised.^183^ Gao *et al.* carried out a structural approach to design a peptide mimicking the antitoxin TplEi to free the toxic TplE based on the success of the approaches described above with MoxXT and VapBC systems.^147,148,165,184^

**Fig. 18:**
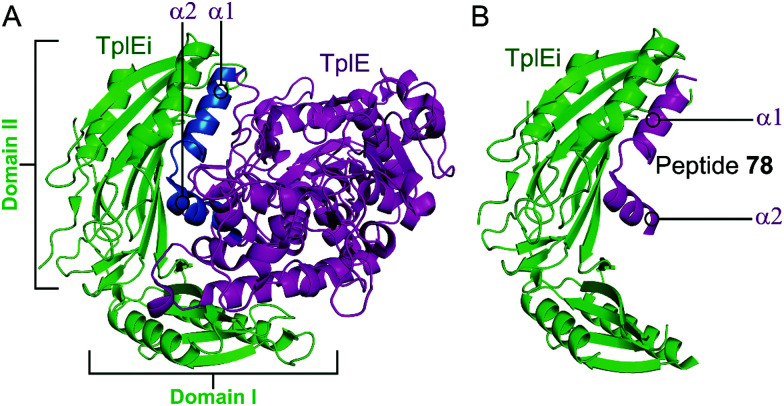
(A) Crystal structure (PDB: 4R1D)^183^ of *P. aeruginosa* TplEi (green) in complex with *P. aeruginosa* TplE (magenta). The antitoxin TplEi can be subdivided in two domains (I and II), with TplE helices α1 and α2 (blue) shown to interact with domain II. (B) Crystal structure (PDB: 5H7Y)^184^ of *P. aeruginosa* TplE-derived L-peptide **78** (magenta) bound to *P. aeruginosa* TplEi (green).

Residues 82–108 of TplE were identified as the binding crucial region with TplEi composed of α1- and α2-helices linked with a disordered loop which binds to a negatively charged groove in TplEi (Fig. 18).^184^ The authors termed this the ‘L’ peptide (**78**) and, using isothermal calorimetry, identified the TplEi–**78** interaction to have tighter binding than the wild type complex (*K*_d_ = 125 nM, Table 10). *E. coli* toxicity assays demonstrated that cell growth was inhibited in the presence of **78**, with the peptide likely competitively binding to TlpEi, freeing the toxic TplE. Structural analysis identified residue K100 as forming four hydrogen bonds with TplEi and using point mutations of the interacting residues in TplEi identified all to be crucial for the tight interaction. Additionally, **78** with a K100E mutation did not inhibit *E. coli* cell growth as strongly as wildtype **78**.

**Table 10:** Peptide inhibitor of TplE–TplEi TA system

TA complex	Mimicked protein	Mimicked region	Peptide	Peptide sequence (residues)^a^	*K*_d_ (nM)
*TplE–TplEi* ^184^	TplE	α1- and α2- helices	**78**	DDLFASIGALWTWAWRGP**K**ARQELLKAEQVEVDD (82–108)	125

a Key residue K100 is highlighted in bold.

## 4 Conclusion

From a development and regulatory point of view, antibiotics are drugs that can be easily progressed to the market. Well-established and simple animal models are available and new antibiotics do not require superior performance to existing drugs, a requirement that allows introduction of new compounds that tackle antimicrobial resistance.^185^ The main reason the antibiotic development pipeline has been dry for many years is a lack of drug discovery campaigns, as targeting pathogenic bacteria poses specific challenges. Drugs with novel modes of action are required to overcome all bacterial mechanisms (tolerance, persistence and resistance) leading to antibiotic treatment failure.

Over the last few decades, PPIs have emerged as promising drug targets and intensive development has led to the transition of PPI modulators as next-generation therapeutics for *e.g.* cancer treatment into the clinic. Naturally, an abundance of PPIs are also present in bacteria and they are therefore increasingly explored as promising antibiotic targets. Bacterial PPIs, exemplified by those presented in this review, are often involved in essential cellular processes, including division and replication (SSB, β-sliding clamp, FtsZ–ZipA), transcription (RNAP, NusB–NusE) and outer membrane protein biosynthesis (BamA–BamD, Lpt). Toxin–antitoxin systems that act on translation, cell wall synthesis or lipolysis are another rich pool of interactions within bacteria that can be utilized for the development of advanced antibiotics. In addition, a number of crucial bacterial PPIs have been well characterised structurally and biophysically, but no inhibitors of these interactions have been reported so far. As PPIs relating directly to functions in bacteria are often conserved in prokaryotes and do not have close human homologues, it may be possible to develop selective drugs without strong side effects in humans and drugs with broad-spectrum activity against high-priority pathogens. Whilst traditional antibiotics that target enzymes are susceptible to resistance formation, by the occurrence of a single mutation in the drug binding pocket of the enzyme that perturbs drug binding but maintains enzyme activity, a mutation of a single hot-spot residue at a PPI interface would not only disrupt drug binding but also disturb the PPI itself and in turn affect bacterial viability. Such a mutation would most likely not persist so readily in the bacterial population, suggesting that PPI-targeting antibiotics might be less susceptible to resistance mechanisms.

Despite all the possibilities that targeting bacterial PPIs offers for the development of effective antibiotics, there are still major obstacles that must be overcome for successful transition of bacterial PPI inhibitors into the clinic. When exploring bacterial PPIs as new targets, it should be verified that their biological function is crucial enough to cause a bacteriostatic or bactericidal effect. This is an ongoing problem as the validation of an observed antibiotic effect as a direct consequence of the inhibition of the PPI in the bacterial cells is still technically challenging and this data is absent in most studies. Additionally, the binding modes of potential PPI inhibitors should be analysed in detail, *i.e.* if they bind competitively with a protein partner or at an allosteric site, as this might influence the probability of resistance formation. Toxin–antitoxin systems as an emerging class of bacterial PPIs for antibiotic development have their own specific challenges. Most reported approaches that target TA systems aim to liberate the toxin to induce bacterial cell death, either by competitive binding to the antitoxin or designing compounds capable of disrupting the TA PPI without inhibiting the activity of the toxin. However, the release of toxins from certain TA systems has been also suggested to induce persistence in bacteria, and an oppositional strategy has been reported that aims to inhibit the toxin to prevent bacterial persister formation. More detailed biological characterization of TA systems is therefore required to assess the consequences of targeting the toxin or antitoxin and to decide if an antibiotic or anti-persistence development strategy can be pursued.

A crucial factor when targeting bacterial PPIs is also the choice of the structural type of the inhibitor. Many PPIs possess binding pockets formed by clusters of hot-spot residues that can be targeted by small molecules.^10^ Inhibitory small molecule compounds have been discovered by *in silico* and high throughput screening, fragment-based lead discovery and rational, structure-based design and are further optimized by intensive SAR development. Small molecule inhibitors have the advantage of high biological stability, potential oral bioavailability and inexpensive synthesis, but the selectivity of small molecule inhibitors for their target proteins needs to be carefully examined. Peptides might be especially useful for bacterial PPIs in which hot-spot residues are distributed over a larger interaction interface due to their potential high affinity and selectivity.^186^ Guided by structural information, linear peptides are often designed as first step to probe the inhibition of a PPI, but their low biological stability necessitates further development into cyclic analogues or peptidomimetics, which may be able to compete with the bioavailability of small molecules. In addition to rational design, HTS using cyclic peptide libraries is increasingly used to identify more drug-like peptidic inhibitors of target PPIs.

One of the biggest challenges, however, that both small molecule and peptidic inhibitors face as potential antibiotics is uptake into bacteria to reach the PPI target. Penetration into Gram-positive bacteria is often achievable, as their main permeability barrier is the plasma membrane which can be penetrated by hydrophobic molecules. In contrast, uptake into Gram-negative bacteria is a major challenge. The lipopolysaccharide (LPS)-rich, anionic outer membrane prevents entry of hydrophobic compounds, while the inner phospholipid bilayer membrane hinders hydrophilic molecules, creating the need for amphipathic compounds. In addition, both membranes are rich in multidrug-resistance pumps that are highly effective in shuttling out undesired compounds and lateral therapy might be required to block these. As cytosolic targets are difficult to reach in Gram-negative bacteria, periplasmic targets or outer membrane PPI targets, such as Fts protein interactions in cell division, can be more attractive. Small molecule inhibitors have traditionally fared better than peptide-based molecules, however, modern cyclic peptides are increasingly able to rival the cell penetration of small molecules.

Overall, the multitude of PPIs specific to bacteria makes them promising targets for antibiotic development and more detailed understanding of the structural features of bacterial PPIs and their exact roles in bacterial biology is highly desired. In combination with the further development of structurally diverse drug compounds with optimized pharmacokinetic and bacterial cell uptake properties, this is expected to pave the way for antibiotics with novel modes of action, much needed in the fight against multidrug-resistant and multidrug-tolerant pathogens.

## Conflicts of interest

There are no conflicts of interest to declare.

## Supplementary Material
